# Auditory and Semantic Processing of Speech‐in‐Noise in Autism: A Behavioral and EEG Study

**DOI:** 10.1002/aur.70097

**Published:** 2025-08-04

**Authors:** Jiayin Li, Maleeha Sujawal, Zivile Bernotaite, Ian Cunnings, Fang Liu

**Affiliations:** ^1^ School of Psychology and Clinical Language Sciences University of Reading Reading UK

**Keywords:** autism, N400, neural tracking, speech‐in‐noise, temporal response functions

## Abstract

Autistic individuals often struggle to recognize speech in noisy environments, but the neural mechanisms behind these challenges remain unclear. Effective speech‐in‐noise (SiN) processing relies on auditory processing, which tracks target sounds amidst noise, and semantic processing, which further integrates relevant acoustic information to derive meaning. This study examined these two processes in autism. Thirty‐one autistic and 31 non‐autistic adults completed a sentence judgment task under three conditions: quiet, babble noise, and competing speech. Auditory processing was measured using EEG‐derived temporal response functions (TRFs), which tracked how the brain follows speech sounds, while semantic processing was assessed via behavioral accuracy and the N400 component, a neural marker of semantic processing. Autistic participants showed reduced TRF responses and delayed N400 onset, indicating less efficient auditory processing and slower semantic processing, despite similar N400 amplitude and behavioral performance. Moreover, non‐autistic participants demonstrated a trade‐off between auditory and semantic processing resources. In the competing speech condition, they showed enhanced semantic integration but reduced neural tracking of auditory information when managing linguistic competition introduced by intelligible speech noise. In contrast, the autistic group showed no modulation of neural responses, suggesting reduced flexibility in adjusting auditory and semantic demands. These findings highlight distinct neural processing patterns in autistic individuals during SiN tasks, providing new insights into how atypical auditory and semantic processing shape SiN perception in autism.

**Summary**

- This study examined how the brain processes speech in noisy environments.
- We found that autistic individuals had reduced and slower brain responses to sounds and meanings.
- Unlike non‐autistic participants, whose brain activity adjusted to different types of background noise, autistic participants showed no such modulation.
- Despite these neural differences, autistic individuals performed as accurately as their non‐autistic peers in judging semantic congruency in the behavioral task.
- These findings provide insights into how autistic individuals navigate complex auditory environments and may inform the development of better communication support in noisy settings.

## 1 Introduction

Recognizing speech in noisy environments, a process known as speech‐in‐noise (SiN) processing, is a complex task influenced by both auditory and cognitive interference from competing sounds (Bronkhorst 2000). Background noise can physically mask speech signals, obscuring key acoustic features and making perception more difficult. This challenge increases when the background contains intelligible speech with similar vocal characteristics, which introduces additional cognitive interference and makes it harder to focus on the target signal (Başkent and Gaudrain 2016; Brungart 2001).

For autistic individuals, these difficulties can be even more pronounced due to atypical auditory and cognitive profile (O'Connor 2012; Ouimet et al. 2012). Previous research on SiN recognition in autism has predominantly focused on auditory processing difficulties, such as challenges in utilizing temporal dips (Alcántara et al. 2004; Groen et al. 2009). Autistic participants are less able to use these brief reductions in noise intensity to enhance target speech recognition. These difficulties extend to continuous noise without temporal dips, particularly under stricter recognition criteria (Schelinski and Von Kriegstein 2020). In multi‐speaker scenarios, autistic listeners experience difficulties in using speaker‐relevant cues, such as spatial or vocal features, to enhance speech separation (DePape et al. 2012; Schaeffer et al. 2023). Additionally, atypical auditory processing in autism is compounded by differences in higher‐order cognitive functions, including verbal abilities (Ruiz Callejo et al. 2023; Russo et al. 2009), attentional control (Emmons et al. 2022), and the integration of auditory information (Lepistö et al. 2009). Neuroimaging studies provide further insights into the neural mechanisms underlying these auditory processing difficulties. Impairments in sensory control have been linked to reduced neural responses in the inferior frontal gyrus under noisy conditions, suggesting disrupted top‐down modulation (Schelinski and Von Kriegstein 2023). Heightened activity in the speech‐processing cortex during SiN tasks indicates compensatory mechanisms for managing auditory challenges (Hernandez et al. 2020). Additionally, increased recruitment of neural resources regardless of task difficulty points to inflexible resource allocation in autism (Mamashli et al. 2017).

Collectively, these findings highlight both auditory difficulties and cognitive challenges during SiN processing in autism. However, no studies have examined SiN recognition in autism with a combined focus on both auditory and semantic processing, despite the crucial role each plays in successful comprehension. To test this, we used electroencephalography (EEG) to examine both auditory and semantic processing in autistic and non‐autistic individuals. Our study builds on Song et al. (2020), who explored the effects of competing speech and babble noise on speech perception. Their findings revealed a significant trade‐off between auditory and semantic processing in non‐autistic listeners. Compared to the unintelligible babble masker, the intelligible speech masker resulted in amplified N400 amplitudes, indicating greater reliance on semantic processing. However, this increased semantic effort was accompanied by less accurate neural tracking of the target speech, suggesting reduced auditory processing. These results support the idea that cognitive resources are limited and dynamically allocated, with greater engagement in semantic processing diminishing resources available for auditory processing. When speech is degraded by noise, skilled listeners rely more on semantic context to compensate for lost acoustic information, thereby facilitating comprehension (Bilger et al. 1984; Kalikow et al. 1977).

The present study builds on this framework to examine whether autistic individuals adopt a similar compensatory strategy during SiN processing. Following Song et al. (2020), we employed a semantic congruency task across three listening conditions: quiet, single‐talker speech noise, and babble noise. To investigate auditory processing, we measured neural tracking of speech envelopes, which capture continuous amplitude fluctuations in speech. Neural tracking reflects the brain's ability to synchronize with rhythmic external stimuli, such as speech (Brodbeck and Simon 2020; Ding and Simon 2012). Neural tracking was estimated using a machine learning approach to predict neural responses from speech envelopes, known as forward modeling (Crosse et al. 2016, 2021). Compared to backward modeling, which reconstructs the stimulus from neural data (Song et al. 2020), forward modeling offers greater insight into the temporal dynamics of speech processing. This approach allows us to examine speech encoding over time (Holdgraf et al. 2017), making it particularly suited for examining the time‐resolved neural processes involved in speech perception under noisy conditions (Ding and Simon 2013; Gillis et al. 2022; Yasmin et al. 2023; Zhang et al. 2023). From this modeling, we obtained the temporal response function (TRF) and focused on P1, N1, and P2 responses. These components closely correspond to auditory evoked potentials (AEPs) and are thought to reflect different auditory processing stages. For example, P1 is associated with early acoustic encoding, while N1–P2 is linked to attention and speech intelligibility (Chen et al. 2023; Di Liberto et al. 2015; Muncke et al. 2022; Orf et al. 2023). Such temporally specific information is not accessible through backward modeling, which provides a single global measure of decoding accuracy but lacks interpretable component‐level resolution.

Although no previous studies have examined TRF components in autistic individuals during SiN tasks, the well‐documented auditory processing difficulties in noise led us to hypothesize that autistic participants would exhibit reduced P1–N1–P2 responses across all conditions. This hypothesis is further supported by findings of atypical neural entrainment in autism in quiet environments (Jochaut et al. 2015), suggesting difficulties in synchronizing brain activity with speech. Additionally, AEP studies have reported atypical P1–N1–P2 responses in autism, indicating reduced cortical responsiveness to acoustic input (O'Connor 2012; Schwartz et al. 2023).

We evaluated semantic processing through both behavioral judgments of semantic violations within the semantic congruency task and corresponding neural responses. Autistic individuals often exhibit atypical cortical response to semantic information even without the presence of noise. This has been investigated using the N400, an ERP component widely recognized as a neural marker of lexical‐semantic processing (Kutas and Hillyard 1980). Typically, N400 amplitudes are larger for less predictable or incongruent words, reflecting greater difficulty in resolving meaning (Hagoort 2008; Osterhout and Holcomb 1992). However, N400 responses are also influenced by individual differences in cognitive and language abilities, and considerable variability has been observed within the autistic population. Autistic individuals—particularly children with poor verbal abilities—often exhibit reduced, delayed, or atypically distributed N400 compared to their non‐autistic peers, suggesting difficulties with semantic integration (Coderre et al. 2017; Fishman et al. 2011; Pijnacker et al. 2010). In contrast, studies focusing on autistic individuals with stronger verbal abilities have reported relatively typical patterns of semantic processing (DiStefano et al. 2019; Henderson et al. 2011; McCleery et al. 2010). Given previous findings of attenuated N400 responses in quiet conditions, we hypothesized that autistic participants would exhibit reduced and delayed N400 responses to SiN stimuli, indicating challenges in semantic integration in noisy environments.

Considering the effect of masker types, we also hypothesized that masker intelligibility would impact the trade‐offs between auditory and semantic processing. For non‐autistic participants, we expected stronger N400 and weaker TRF responses in the intelligible speech masker condition compared to the unintelligible babble condition, consistent with Song et al. (2020). In contrast, we predicted that autistic participants would show less differentiation between conditions of varying intelligibility, reflecting reduced top‐down modulation during SiN processing.

Finally, prior research has identified a range of cognitive factors that may contribute to variability in SiN perception among autistic individuals. For example, temporal processing difficulties have been found to correlate more closely with language ability than with autism diagnosis per se (DePape et al. 2012; Bhatara et al. 2013). Similarly, verbal IQ may influence performance at an individual level, even when group‐level differences are not observed (Ruiz Callejo et al. 2023). Difficulties with selective auditory attention have also been reported in autism (Emmons et al. 2022; Lau et al. 2023). Taken together, these findings highlight the complex and multifactorial nature of SiN perception in autism. Based on this evidence, and in line with recent work showing that cognitive abilities can predict neural and behavioral responses to SiN (Ruiz Callejo and Boets 2023), we conducted exploratory correlation analyses to examine potential associations among cognitive abilities, behavioral accuracy, and neural responses.

## 2 Methods

### 2.1 Participants

We recruited 31 autistic and 31 non‐autistic participants, aged 17–47, all of whom were right‐handed native English speakers. Participants passed a hearing screening using an Amplivox manual audiometer, confirming normal hearing in both ears at 25 dB for frequencies of 0.5, 1, 2, and 4 kHz. Both groups had no current speech, language, or communication needs. Autistic participants had diagnoses confirmed by professional clinicians and supported by clinical reports. Non‐autistic participants reported no personal or family history of autism, and this was further supported by their scores on the Autism Spectrum Quotient (AQ) (Baron‐Cohen et al. 2001), all of which were below the cut‐off of 32.

We measured cognitive abilities that may influence SiN processing (Gordon‐Salant and Cole 2016; Heinrich 2021). Nonverbal IQ was measured using Raven's Standard Progressive Matrices (Raven and Court 1998), while receptive vocabulary, a proxy for verbal IQ, was assessed using the Receptive One‐Word Picture Vocabulary Test‐Fourth Edition (ROWPVT‐4; Martin and Brownell 2011). Verbal short‐term memory was evaluated with the digit span task (Wechsler et al. 2003). Participants also completed a musical training questionnaire (Pfordresher and Halpern 2013), which recorded years of formal training across various instruments. Additionally, auditory‐related traits were measured using the Auditory Attention and Discomfort Questionnaire (Dunlop et al. 2016), which assessed difficulties with auditory attention in noisy environments and sensitivity to auditory stimuli in daily life.

Demographic and cognitive data are summarized in Table 1. Welch's two‐sample *t*‐tests showed no significant differences between autistic and non‐autistic groups in chronological age, musical training background, receptive vocabulary, nonverbal reasoning ability, or verbal short‐term memory. However, the autistic group scored significantly higher on the AQ, reflecting elevated autistic traits, and reported greater auditory attention difficulties and discomfort.

**TABLE 1:** Characteristics of the autistic (*n* = 31) and non‐autistic groups (*n* = 31).

Variables	Autistic *M* (SD)	Non‐autistic *M* (SD)	*W*	*p*	Rank‐biserial correlation
Gender (female:male)	22:9	26:5			
Age	25.73 (7.89)	25.78 (7.83)	484.0	0.97	0.01
Musical training	4.02 (5.61)	6.39 (7.02)	384.0	0.16	−0.20
Nonverbal reasoning (RSPM raw core)	53.87 (3.59)	54.39 (3.61)	441.0	0.58	−0.08
Nonverbal reasoning (RSPM percentile)	49.03 (23.96)	52.74 (29.32)	458.5	0.75	−0.05
Receptive vocabulary (ROWPVT‐4 raw score)	167.16 (10.40)	170.03 (8.35)	429.5	0.48	−0.11
Receptive vocabulary (ROWPVT‐4 standard score)	109.26 (15.92)	113.10 (14.69)	420.5	0.40	−0.10
Digit span	7.07 (1.61)	7.07 (1.03)	464.0	0.82	−0.03
Auditory attention difficulty	38.58 (10.03)	24.74 (9.47)	811.5	**< 0.01**	0.69
Auditory discomfort	60.94 (9.76)	43.81 (10.44)	855.5	**< 0.01**	0.78
Autistic traits (AQ)	38.29 (6.62)	17.13 (8.49)	935.0	**< 0.01**	0.95

*Note:* The *p*‐values of significant fixed effects are presented in bold.

The study was approved by the University Research Ethics Committee, and all participants provided written informed consent. Participants received financial compensation. Student participants recruited from the psychology participant pool were awarded course credits.

### 2.2 Stimuli and Apparatus

The target stimuli consisted of 180 sentence pairs with highly constraining contexts. The final word in each sentence was either semantically congruent (e.g., I passed my test and got my driving license) or incongruent with the preceding context (e.g., I passed my test and got my driving discount).

Semantically incongruent sentences were expected to elicit larger N400 amplitudes than congruent sentences, reflecting the modulation of N400 responses during semantic integration. Sentences were drawn from a validated set developed by Stringer and Iverson (2020). Each sentence contained 5–10 words (5–13 syllables) and was recorded by a female native speaker of Southern British English.

The maskers were adopted from Song et al. (2020). The single‐talker speech masker consisted of recordings of English stories read by the same speaker as the target sentences. The babble masker was created by processing the speech masker: the recordings were segmented and randomly rearranged to ensure acoustic consistency while making the speech semantically unintelligible. The signal‐to‐noise ratio was set to 0 dB, based on a pilot study (see Supporting Information for details of the maskers and the pilot study).

Participants completed the experiment using E‐Prime 3.0 software in a soundproof booth. Audio stimuli were presented binaurally through Etymotic ER‐1 earphones at 67 dB sound pressure level. Participants judged sentence acceptability while disregarding background noise. Prior to the experiment, participants completed three practice items per condition to ensure understanding of the task. During each trial, an audio file was played alongside a fixation cross displayed on the screen. After a silent interval (1.5–1.7 s), participants judged the sentence as acceptable or unacceptable.

The experiment comprised six blocks (two per condition), with 60 trials per block lasting 5–6 min. Sentences of varying congruency were randomly mixed, and block order was randomized. To minimize context effects, three experimental lists were created, with conditions counterbalanced across lists. Lists were randomly assigned to participants. Self‐paced breaks between blocks were provided to reduce fatigue.

### 2.3 EEG Recording and Pre‐Processing

EEG data were recorded using a Biosemi Active Two system with 64 Ag/AgCl electrodes and six external electrodes (left/right mastoids and vertical/horizontal electrooculography). Signals were recorded at a sampling rate of 2048 Hz without referencing, and electrode impedances were kept below 25 kΩ. Triggers marking the onset of target words were recorded with the EEG.

Data pre‐processing was performed in EEGLAB (Delorme and Makeig 2004) within Matlab R2018b. For TRF analysis, EEG signals were band‐pass filtered between 1 and 8 Hz using a zero‐phase Butterworth filter to isolate low‐frequency activity (Ahissar et al. 2001; Luo and Poeppel 2007). The data were then downsampled to 64 Hz for computational efficiency. The speech envelope, used as the input acoustic feature for TRF modeling, was extracted via the Hilbert transform, downsampled to 64 Hz, and normalized with the EEG data (mean‐subtracted and standardized). EEG trials were precisely aligned with stimulus segments to ensure matching data lengths.

For N400 analysis, signals were low‐pass filtered at 40 Hz using a zero‐phase Butterworth filter, downsampled to 256 Hz, and re‐referenced to the average of the mastoids. Data were segmented into epochs ranging from −200 to 800 ms relative to the target word onset and baseline‐corrected using the pre‐stimulus interval (−200 to 0 ms). Bad channels were manually identified and interpolated. Independent Component Analysis was performed using the runica algorithm implemented in EEGLAB to decompose the continuous EEG data into independent components. Artefactual components were identified and rejected based on both automatic classification and manual inspection. Specifically, we used the ICLabel plugin (Pion‐Tonachini et al. 2019) to estimate the probability that each component reflected neural activity, eye movements, muscle activity, or other sources of noise. Components classified as “eye” or “muscle” with a probability of at least 75% were considered candidates for removal. All flagged components were further examined manually, with particular attention to topography, time series, and power spectrum characteristics. On average, 3.87 trials per participant (approximately 1% of all trials) were excluded due to artifacts in the autistic group, and 2.00 trials per participant (approximately 0.6% of all trials) were excluded in the non‐autistic group. A Wilcoxon rank‐sum test showed no significant group difference in the number of excluded trials (*W* = 577.5, *p* = 0.154), with a small, non‐significant effect size (*r* = 0.20, 95% CI [−0.08, 0.46]), indicating comparable trial rejection rates across groups.

### 2.4 EEG Data Analysis

#### 2.4.1 TRF Modeling

We conducted TRF modeling using the mTRF toolbox (Crosse et al. 2016). Models were fitted with a time‐lag window of [−100, 400 ms] to capture neural responses at latencies between 0 and 300 ms (Di Liberto et al. 2018). Separate models were created for each condition and group, with ridge regression and regularization (*λ*) employed to prevent overfitting. An individual, subject‐specific approach was used to train and cross‐validate the TRF models, following the procedures outlined in Crosse et al. (2021), to estimate the TRF that best fits each participant's neural responses. Optimal *λ* values were selected via 10‐fold cross‐validation, testing a range ([10^−6^, …, 10^4^]) and selecting the *λ* yielding the highest average Pearson correlation between predicted and actual EEG signals (Zion Golumbic et al. 2013). The resulting TRF waveforms represent how the EEG signal at each electrode changes in response to a unit change in the speech stimulus envelope. Pearson correlation coefficient *r* between the predicted and recorded EEG signals was also calculated to evaluate the overall strength of neural tracking.

#### 2.4.2 Cluster‐Based Permutation Tests

For both auditory (TRF) and semantic (N400) processing, we applied cluster‐based permutation tests (CBPT, Maris and Oostenveld 2007) using the FieldTrip toolbox (Oostenveld et al. 2011). Paired *t*‐tests were conducted at each electrode and time point to assess differences between conditions or groups. To identify candidate clusters, a two‐sided threshold of *p* < 0.05 was applied to the resulting sample‐level *t*‐tests, and spatiotemporally adjacent significant data points were grouped into clusters. For each cluster, a cluster‐level statistic was calculated as the sum of the *t*‐values within the cluster. Statistical significance was assessed using a two‐sided Monte Carlo permutation test with 1000 random permutations of condition labels. Clusters were considered significant if their cluster‐level statistic fell within the top or bottom 2.5% of the permutation distribution, corresponding to an overall corrected alpha level of 0.05. For tests conducted separately across group and condition, Bonferroni correction was applied to control for multiple comparisons.

This non‐parametric approach is especially useful for identifying spatiotemporally extended effects without imposing strong a priori constraints on when or where such effects might occur. However, CBPTs are inherently limited to pairwise comparisons and do not provide reliable estimates of effect latency or precise topography (Sassenhagen and Draschkow 2019). To address these limitations, we complemented CBPTs with additional latency analyses and targeted statistical testing using linear mixed‐effects models, allowing us to quantify amplitude and latency differences and assess interactions between group and condition effects with greater precision.

#### 2.4.3 Latency Analysis

Latency detection methods were chosen to match the temporal characteristics of each ERP/TRF component. For early TRF components (P1, N1, and P2), which are characterized by sharp, time‐locked peaks, we used traditional peak latency detection within predefined windows (Luck 2005). In contrast, N400 latency was estimated using the fractional area latency (FAL) algorithm implemented in ERPLAB, a method recommended for broader and more variable components to provide robust and reliable estimates of onset latency (Lopez‐Calderon and Luck 2014).

Latency windows for each TRF component were defined based on the mean and standard deviation (SD) of observed peaks across participants. Each window was set as mean ± 2 SD to capture approximately 95% of latency variability and was visually validated against grand‐averaged waveforms to ensure alignment with observed peak distributions. Within these validated windows, peak latency and amplitude were identified for each participant and condition.

The onset latency of the N400 effect was estimated using the fractional area latency (FAL) method, following the guidelines by Lopez‐Calderon and Luck (2014). We computed the area under the N400 difference waveform (incongruent minus congruent) within the 200–500 ms time window at posterior midline electrodes (Cz, CPz, and Pz), and identified the time point at which 20% of the total area was reached. This measure was calculated separately for each participant and condition.

### 2.5 Statistical Analysis

Analyses were conducted in R (version 4.1.2; R Core Team 2022). Linear mixed‐effects models (LMMs) were fitted for TRF and N400 data including component amplitudes, latencies, and Pearson correlation (*r*). Generalized linear mixed‐effects models (GLMMs) were constructed for behavioral accuracy (binary outcome), with the BOBYQA optimizer applied to improve convergence. All models were constructed using the lme4 package (Bates et al. 2015). Models compared performance across background conditions using two masker contrasts: (1) baseline (no maskers) versus masker conditions (babble, speech) and (2) babble versus speech maskers.

Fixed effects included group (autistic = 1/2, non‐autistic = −1/2), masker (contrast 1: babble = 1/3, speech = 1/3, baseline = −2/3; contrast 2: babble = 1/2, speech = −1/2, baseline = 0), and their interactions. For ERP models, sentence type (congruent = 1/2, incongruent = −1/2) was included as an additional fixed effect. Model selection followed the recommendations of Barr et al. (2013). Initial models were fitted with a maximal random‐effects structure, including random intercepts for participants and by‐participant random slopes for within‐subject predictors. For the behavioral data, the maximal model also included by‐trial random effects (random intercepts and slopes). In contrast, TRF and N400 data were grand‐averaged across trials for each condition and participant prior to statistical analysis to reduce trial‐level noise; therefore, trial‐level variability was not modeled, and random effects for trials were not included.

When maximal models failed to converge, the random‐effects structure was simplified in a stepwise manner: (1) by removing correlations between random effects, and (2) by incrementally adding random slopes to an intercept‐only model to identify the most parsimonious structure that captured meaningful variance. As models included group effect as a between‐subject factor, random intercepts for participants were retained in all models to account for individual baseline differences. At each step, likelihood ratio tests were used to compare models and retain only random effects that significantly improved model fit. Fixed effects and interactions were tested using likelihood ratio tests by comparing the final model to nested models with specific fixed effect removed. Significant interactions were followed up with simple effects analyses by subsetting the data and refitting the model. Bonferroni correction was applied to control for multiple comparisons, with the alpha level set at 0.025 for main and interaction effects, and 0.0125 for simple effects. For effect sizes, partial eta‐squared (*η*_*p*_^2^) was computed for each fixed effect in LMMs with ≥ 0.01, ≥ 0.09, and ≥ 0.25 interpreted as small, medium, and large effects, respectively (Cohen et al. 2013). For GLMMs with binary outcomes, odds ratios (ORs) were calculated by exponentiating the model coefficients. An OR of 1 indicates no effect, while values farther from 1 (either above or below) reflect stronger effects.

## 3 Results

### 3.1 Behavioral Results

**FIGURE 1:**
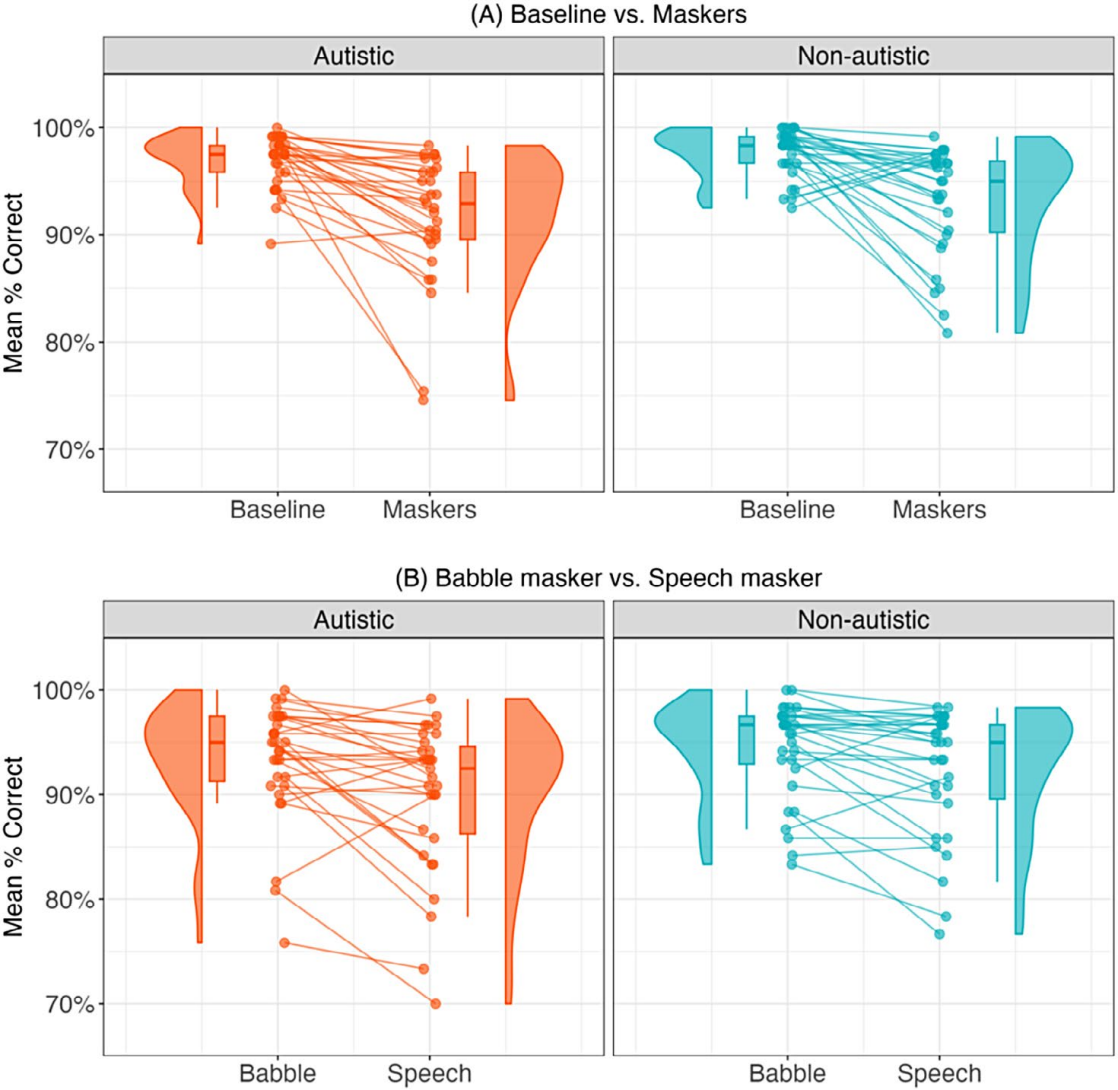
Performance accuracy across conditions for autistic and non‐autistic groups. (A) Compares baseline to masker conditions (average of babble and speech maskers). (B) Compares babble to speech maskers. Violin plots with embedded box plots show the distribution of mean percentage accuracy, with individual data points connected to illustrate within‐subject differences.

Figure 1 shows the accuracy for both masker contrasts across groups. Overall, both groups performed well on the task, particularly in the baseline condition, where ceiling performance was observed. The GLMM analysis (Table 2) revealed significant main effects of both masker contrasts. Behavioral accuracy was higher in the baseline condition compared to the masker conditions (baseline: *M*_NAS_ = 97.7%, SD_NAS_ = 14.9%; *M*_AS_ = 96.9%, SD_AS_ = 17.2%. Maskers: *M*_NAS_ = 93.3%, SD_NAS_ = 25.1%; *M*_AS_ = 91.7%, SD_AS_ = 27.7%). Additionally, both groups performed better in the babble condition (*M*_NAS_ = 94.4%, SD_NAS_ = 23.1%; *M*_AS_ = 93.3%, SD_AS_ = 24.9%) than in the speech condition (*M*_NAS_ = 92.2%, SD_NAS_ = 26.9%; *M*_AS_ = 90.0%, SD_AS_ = 30.0%). No significant group effect or interactions were found, indicating comparable accuracy rates across masker conditions.

**TABLE 2:** Results of the GLMM for behavioral data.

Fixed effects	Est/beta	SE	*z*	*χ* ^2^	*p*	OR
(Intercept)	3.51	0.11	31.22	—	—	—
Group	−0.31	0.19	−1.62	2.54	0.112	0.73
Masker‐1	−1.10	0.12	−9.15	58.85	**< 0.001**	0.33
Masker‐2	0.46	0.09	5.10	21.56	**< 0.001**	1.58
Group × Masker‐1	0.07	0.22	0.32	0.10	0.752	1.07
Group × Masker‐2	0.13	0.16	0.81	0.61	0.434	1.14

*Note:* The *p*‐values of significant fixed effects are presented in bold. Model structure: glmer(Accuracy ~1 + Group × Masker‐1 + Group × Masker‐2 + (1 + Masker‐1 + Masker‐2 | Subject) + (1 | Trial)).

Abbreviation: OR, odds ratios.

### 3.2 TRF Results

We conducted cluster‐based permutation tests (CBPTs) to identify statistically significant spatiotemporal clusters within a 0–300 ms time window. As CBPTs are limited to pairwise comparisons, we adopted a structured analysis plan to match our theoretical contrasts of interest and to remain as consistent as possible with our follow‐up LMMs.

Initially, we explored the main effects of group across conditions, but no significant clusters emerged. We suspect this may be due to variability in the latency and polarity of the P1–N1–P2 complex across groups and conditions, which can dilute effects when aggregated. Therefore, we performed separate CBPTs within each group and condition and applied Bonferroni correction to account for multiple comparisons (McClannahan et al. 2019). This approach allowed us to better capture condition‐specific or group‐specific TRF effects without assuming consistent timing or morphology across all comparisons.

For the group effect, three tests were conducted (one per condition), resulting in a corrected alpha of 0.05/3 ≈ 0.017. For the condition effect, we examined two theoretically motivated contrasts within each group: (1) baseline versus maskers and (2) babble versus speech, resulting in four comparisons in total and a corrected alpha of 0.05/4 = 0.0125. These two contrasts were selected to remain consistent with our LMMs, which were designed to address the same comparisons, rather than testing each condition individually.

**FIGURE 2:**
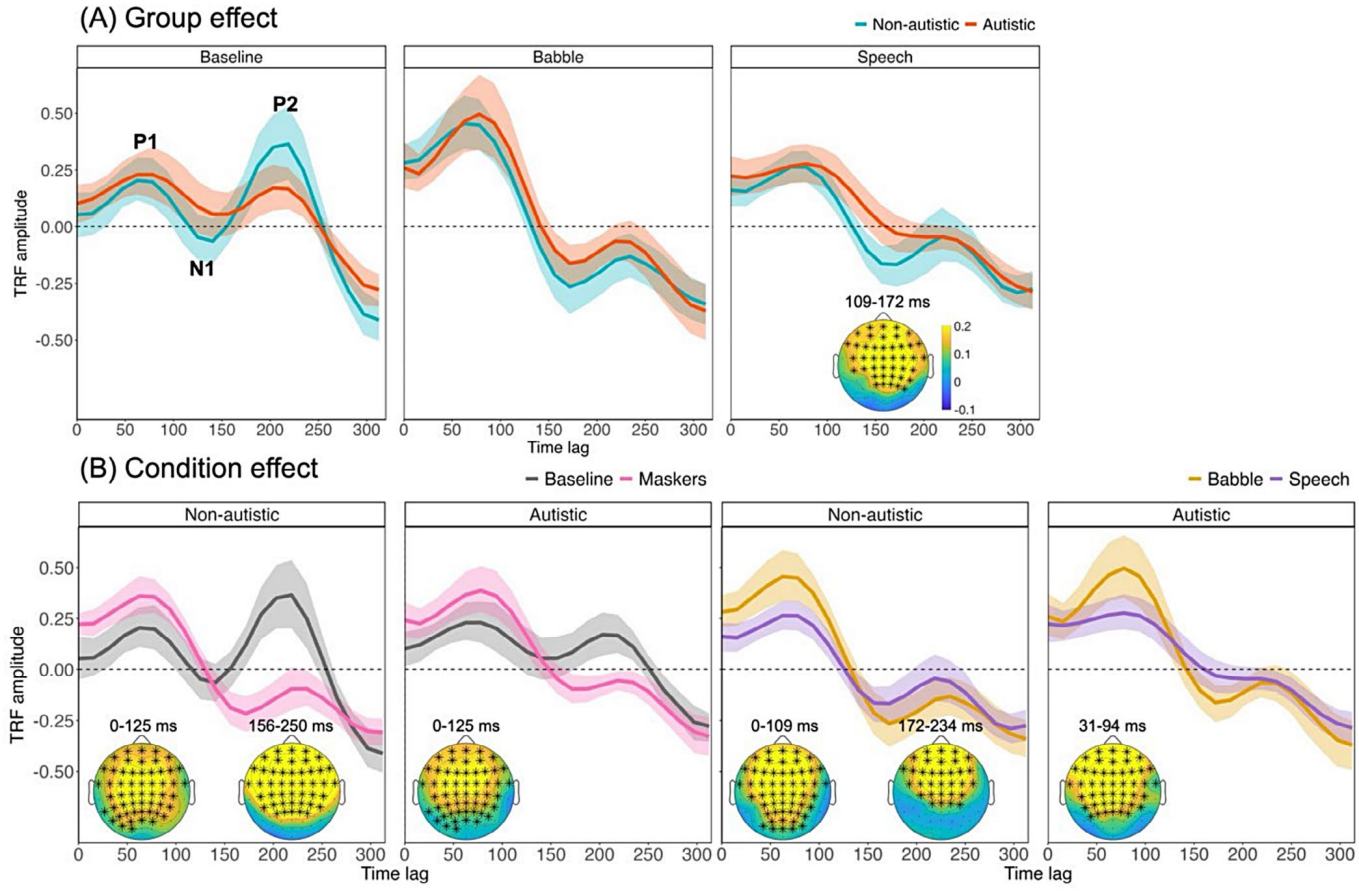
Results of the cluster‐based permutation tests for TRF group and condition effects. Each panel includes line plots showing mean TRF waveforms, and topographic maps highlighting scalp regions and time windows where significant clusters were identified. Asterisks indicate the scalp locations of these clusters, with the corresponding time windows labeled next to each map. The maps reflect the absolute amplitude differences (in μV) between the compared groups or conditions, with the color scale indicating the magnitude of the differences. (A) Group comparisons between autistic and non‐autistic participants within each listening condition (baseline, babble, and speech). Approximate peak of the P1, N1, and P2 components are labeled in the baseline waveform for reference. (B) Condition comparisons within each group. Left panels compare baseline to masker conditions (babble and speech combined); right panels compare babble to speech.

Figure 2A shows the results of cluster‐based permutation tests examining group differences within each condition. The waveforms illustrate the latency, amplitude, and morphology of TRF components. In the baseline condition, the P1, N1, and P2 peaks in the non‐autistic group are clearly identifiable (as marked in the figure), closely resembling traditional auditory evoked potentials (AEPs) in both latency and polarity. In the speech masker condition, a significant cluster was observed between 109 and 172 ms (*p* = 0.010), as shown in the topographic map, with activity primarily distributed over fronto‐central electrodes. This cluster falls within the expected N1 time window and reflects stronger neural tracking of the speech envelope in the non‐autistic group compared to the autistic group.

Figure 2B presents the results of condition effects within each group. In the early P1 time window, both groups exhibited significant clusters when comparing baseline to masker conditions (both *p*‐values < 0.001, 0–125 ms), indicating reduced TRF amplitudes in the absence of background noise. In addition, both groups showed significant clusters in the comparison between babble and speech maskers, with reduced TRF responses in the speech condition. For the non‐autistic group, the cluster spanned 0–109 ms (*p* < 0.001), while for the autistic group, the cluster was observed from 31 to 94 ms (*p* < 0.001), both falling within the P1 response window.

In the later N1–P2 time range, a significant cluster was found in the non‐autistic group for the baseline versus masker contrast between 156 and 250 ms (*p* < 0.001), suggesting reduced auditory cortical responses in noisy compared to quiet conditions. This implies that neural tracking of the speech envelope was more robust in the absence of background noise for non‐autistic participants. No corresponding effect was observed in the autistic group, indicating a lack of measurable differentiation between quiet and noisy conditions. For the babble versus speech contrast, a significant cluster in the non‐autistic group was observed between 172 and 234 ms (*p* = 0.002), reflecting stronger TRF responses in the speech condition. No significant differences were found in the autistic group, suggesting comparable auditory tracking responses across masker types.

**FIGURE 3:**
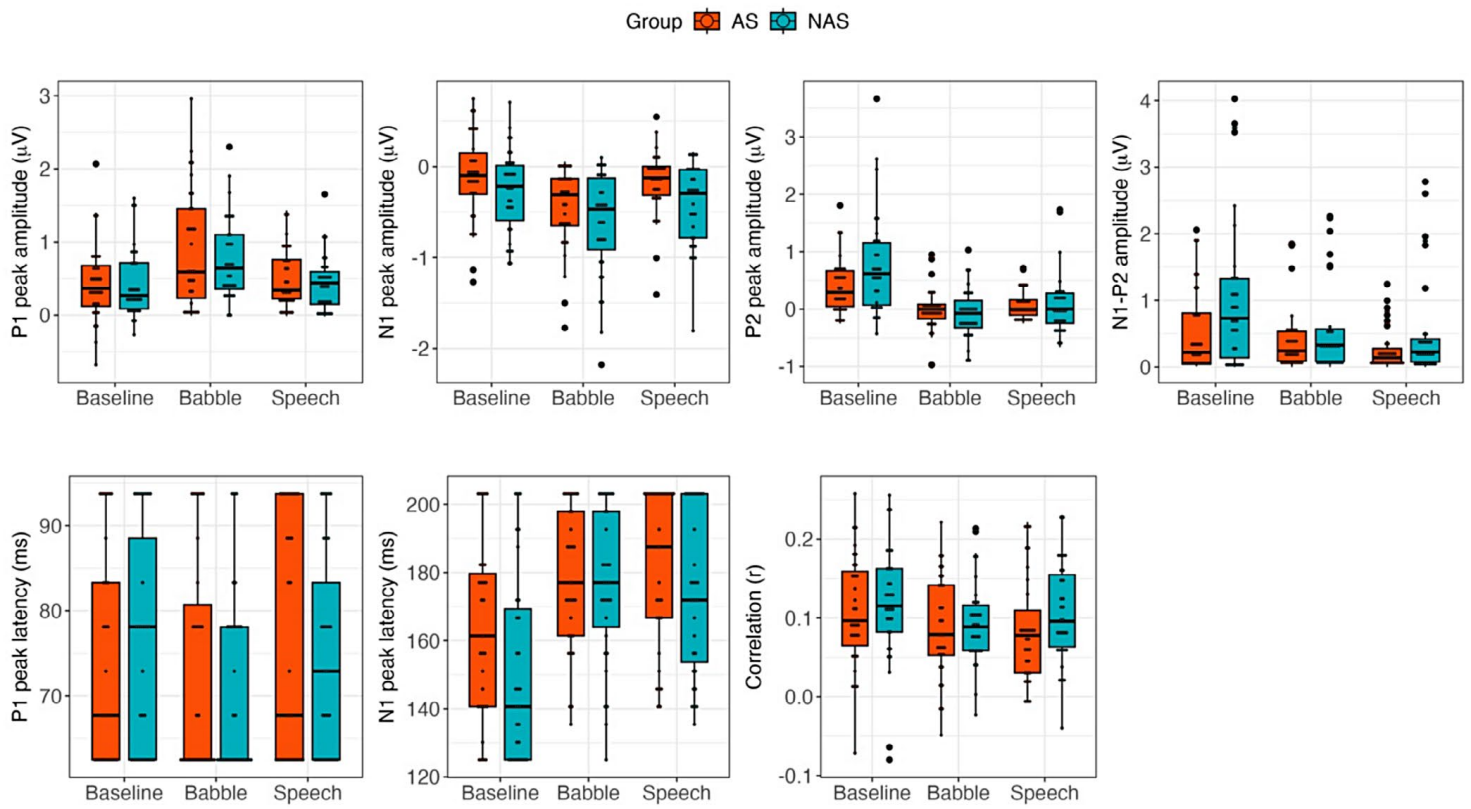
TRF component amplitudes, latencies, and model fit across conditions and groups. Boxplots show peak amplitudes and latencies of the TRF P1, N1 components, as well as the amplitude of P2 and N1–P2 (P2 minus N1), and Pearson correlation coefficients (*r*) between predicted and recorded EEG signals. Data are presented by condition (baseline, babble, and speech) and group (AS, autistic; NAS, non‐autistic).

Since CBPTs could not capture specific TRF components and latency variability, LMMs were conducted to examine P1, N1, and P2 responses separately, focusing on frontal‐central electrodes (AFz, Fz, F1, F2, F3, F4, FCz, FC1, FC2, FC3, FC4, Cz, C1, C2, C3, C4) (Muncke et al. 2022). This approach allowed for a systematic interpretation of how individual TRF components drive the observed differences, providing more precise insights into auditory processing mechanisms. Peak amplitudes and latencies were examined for the P1 and N1 components. For the N1 component, a “larger” response indicates a more negative deflection, reflecting stronger neural activation. For the P2 component, only amplitude was analyzed, as the peak was not reliably distinguishable across conditions and therefore unsuitable for latency analysis. Additionally, because P2 exhibited negative polarity in some conditions, we also examined the amplitude difference between P2 and N1 (P2 minus N1) as a more reliable index of later auditory processing (e.g., Beauducel et al. 2000). Full results are reported in Table 3. Meanwhile, the Pearson correlation coefficient (*r*) between actual and predicted EEG signals was also included in the statistical analysis (see Table 4 for the results). Box plots for all measured variables are shown in Figure 3.

**TABLE 3:** Results of the LMM for TRF component amplitudes and latency.

	Fixed effects	Est/beta	SE	*t*	*χ* ^2^	*p*	*η*_*p*_^2^
P1 amplitude	(Intercept)	0.54	0.05	10.34	—	—	—
	Group	0.06	0.10	0.62	0.38	0.537	0.01
	Masker‐1	0.20	0.05	3.86	13.36	**< 0.001**	0.19
	Masker‐2	0.35	0.06	5.80	26.84	**< 0.001**	0.35
	Group × Masker‐1	0.08	0.10	0.79	0.61	0.433	0.01
	Group × Masker‐2	0.05	0.12	0.45	0.20	0.656	0.00
P1 latency	(Intercept)	74.17	0.98	75.47	—	—	—
	Group	1.79	1.97	0.91	0.82	0.365	0.01
	Masker‐1	−1.95	1.69	−1.15	1.31	0.252	0.02
	Masker‐2	−2.82	1.82	−1.55	2.35	0.125	0.04
	Group × Masker‐1	2.72	3.38	0.81	0.65	0.421	0.01
	Group × Masker‐2	−1.73	3.64	−0.48	0.23	0.635	0.00
N1 amplitude	(Intercept)	−0.32	0.04	−8.31	—	—	—
	Group	0.16	0.08	2.13	4.40	**0.036**	0.07
	Masker‐1	−0.21	0.07	−3.26	9.78	**0.002**	0.15
	Masker‐2	−0.22	0.05	−4.13	15.05	**< 0.001**	0.22
	Group × Masker‐1	0.04	0.13	0.31	0.09	0.759	0.00
	Group × Masker‐2	−0.04	0.11	−0.42	0.17	0.678	0.00
N1 latency	(Intercept)	170.73	2.17	78.53	—	—	—
	Group	7.79	4.35	1.79	3.13	0.077	0.05
	Masker‐1	19.43	2.98	6.52	32.33	**< 0.001**	0.41
	Masker‐2	−1.21	3.01	−0.40	0.16	0.687	0.00
	Group × Masker‐1	−5.75	5.96	−0.96	0.92	0.337	0.01
	Group × Masker‐2	−7.34	6.01	−1.22	1.47	0.225	0.02
P2 amplitude	(Intercept)	0.20	0.05	3.86	—	—	—
	Group	−0.08	0.10	−0.78	0.60	0.439	0.01
	Masker‐1	−0.53	0.06	−8.63	48.95	**< 0.001**	0.55
	Masker‐2	−0.11	0.05	−2.18	4.59	**0.032**	0.07
	Group × Masker‐1	0.35	0.12	2.79	7.35	**0.007**	0.11
	Group × Masker‐2	0.06	0.10	0.66	0.43	0.513	0.01
N1–P2 amplitude	(Intercept)	0.52	0.06	8.20	—	—	—
	Group	−0.24	0.13	−1.93	3.61	0.057	0.06
	Masker‐1	−0.32	0.09	−3.52	11.27	**< 0.001**	0.17
	Masker‐2	0.11	0.07	1.50	2.20	0.138	0.03
	Group × Masker‐1	0.31	0.18	1.67	2.73	0.098	0.04
	Group × Masker‐2	0.11	0.15	0.73	0.53	0.465	0.01

*Note:* The *p*‐values of significant effects are presented in bold. The same model was used for all analyses of amplitude and latency: lmer(Amplitude/Latency ~1 + Group × Masker‐1 + Group × Masker‐2 + (1 + Masker‐1 + Masker‐2 | Subject)).

**TABLE 4:** Results of the LMM for *r* values of TRF modeling.

Fixed effects	Est/beta	SE	*z*	*χ* ^2^	*p*	*η*_*p*_^2^
(Intercept)	3.51	0.11	31.22	—	—	—
Group	−0.31	0.19	−1.62	2.54	0.112	0.01
Masker‐1	−1.10	0.12	−9.15	58.85	**< 0.001**	0.09
Masker‐2	0.46	0.09	5.10	21.56	**< 0.001**	0.00
Group × Masker‐1	0.07	0.22	0.32	0.10	0.752	0.00
Group × Masker‐2	0.13	0.16	0.81	0.61	0.434	0.01

*Note:* The *p*‐values of significant fixed effects are presented in bold. Model structure: lmer(*r*‐value ~1 + Group × Masker‐1 + Group × Masker‐2 + (1 + Masker‐1 + Masker‐2 | Subject)).

#### 3.2.1 P1 Amplitude

There was no significant effect of group or any group × condition interactions. However, both masker contrasts yielded significant main effects. P1 amplitude was reduced in the baseline condition (*M* = 0.40, SD = 0.47) relative to the masker conditions (*M* = 0.60, SD = 0.56). Within the masker conditions, babble noise (*M* = 0.78, SD = 0.65) elicited significantly greater P1 amplitudes than speech maskers (*M* = 0.43, SD = 0.37).

#### 3.2.2 P1 Latency

No significant main effects or interactions emerged for P1 latency.

#### 3.2.3 N1 Amplitude

A significant group effect was found, with non‐autistic participants (*M* = −0.40, SD = 0.49) showing stronger (more negative) N1 responses than autistic participants (*M* = −0.24, SD = 0.43). Additionally, both masker contrasts showed significant main effects. N1 amplitude was stronger in the masker conditions (*M* = −0.39, SD = 0.46) compared to the baseline condition (*M* = −0.18, SD = 0.45). Meanwhile, more negative responses were observed in the babble condition (*M* = −0.50, SD = 0.50) relative to the speech condition (*M* = −0.28, SD = 0.40). No interactions reached significance.

#### 3.2.4 N1 Latency

Group differences in latency were marginal (*p* = 0.078), with autistic participants (*M* = 174.62, SD = 27.24) showing delayed responses compared to non‐autistic participants (*M* = 166.83, SD = 26.70). A significant main effect of condition was observed, with longer latencies in masker conditions (*M* = 177.21, SD = 23.73) than in the baseline (*M* = 157.78, SD = 29.14). No significant interactions were observed.

#### 3.2.5 P2 Amplitude

Significant main effects of both masker contrasts were also detected. The baseline condition (*M* = 0.56, SD = 0.69) elicited larger amplitudes compared to masker conditions (*M* = 0.02, SD = 0.40). Between maskers, the speech condition (*M* = 0.07, SD = 0.40) showed slightly larger responses than babble (*M* = −0.03, SD = 0.40). However, given the variability in N1 across conditions, this result should be interpreted cautiously. A significant interaction between group and baseline‐masker contrast was observed. Post hoc analyses revealed significant baseline‐masker differences in both autistic (*χ* ^2^(1) = 17.73, *p* < 0.001) and non‐autistic groups (*χ* ^2^(1) = 30.05, *p* < 0.001). No group differences were found within either the baseline (*χ* ^2^(1) = 3.34, *p* = 0.067) or masker conditions (*χ* ^2^(1) = 0.18, *p* = 0.671).

#### 3.2.6 N1–P2 Amplitude

A marginal group effect was observed (*p* = 0.057), with stronger N1–P2 responses in the non‐autistic group (*M* = 0.64, SD = 0.82) compared to the autistic group (*M* = 0.39, SD = 0.52). A significant main effect of condition was also present: baseline responses (*M* = 0.73, SD = 0.86) were greater than those under masker conditions (*M* = 0.41, SD = 0.57). No significant interactions were observed.

#### 3.2.7 Neural Tracking Strength (*r*)

There was a significant difference between the baseline and maskers conditions, with greater *r* values in the baseline condition (*M* = 0.11, SD = 0.08) compared to the masker conditions (*M* = 0.09, SD = 0.07). No group differences or interactions were found, indicating comparable tracking strength between groups.

### 3.3 ERP Results

**FIGURE 4:**
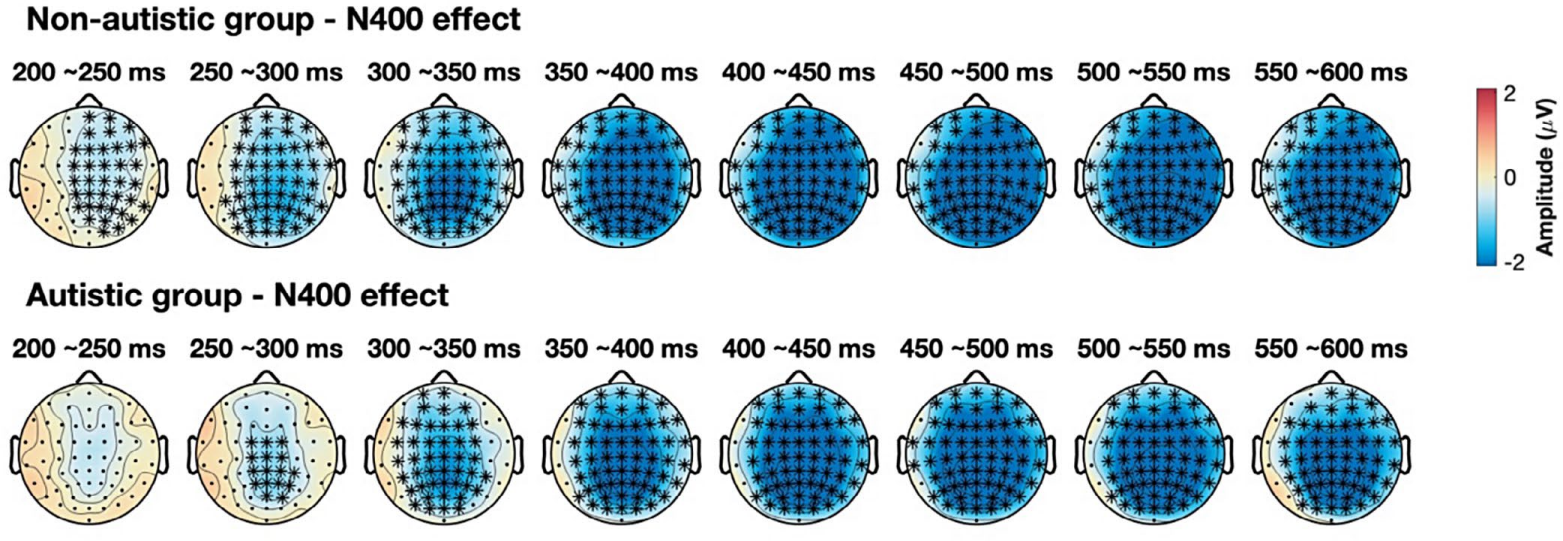
Results of the cluster‐based permutation test of the N400 effect (incongruent–congruent) in each group. Topographic maps display the strength of ERP amplitude difference between incongruent and congruent sentences in 50 ms time bins from 200 to 600 ms for the non‐autistic (top row) and autistic (bottom row) groups. Asterisks indicate the time windows and scalp regions where significant clusters were identified. The maps reflect the value of amplitude differences (in μV), with the color scale indicating the polarity and magnitude of the effect.

Two cluster‐based permutation tests were conducted within the 200–600 ms time window, following the procedure described by Song et al. (2020). We first compared responses to incongruent versus congruent sentences in each group to identify clusters reflecting N400 variation. Significant differences were observed in both groups across all masker conditions (both *p*‐values < 0.001), indicating that both groups showed significant N400 effects. As shown in Figure 4, there was a significant cluster across the scalp between 200 and 600 ms for non‐autistic listeners. In contrast, a significant cluster was found between 250 and 600 ms for autistic listeners, suggesting a delayed onset of the N400 response. This was further verified by a statistical analysis of N400 onset latency (see Section 2.4.3 for the method). The autistic group showed significantly longer latencies than the non‐autistic group, indicating delayed semantic processing (see Table 5 for results). We then examined the effect of noise conditions on the N400 within each group by comparing N400 amplitudes across two masker contrasts: (1) baseline versus noise maskers, and (2) babble versus speech masker. After applying Bonferroni correction for multiple comparisons, no significant clusters were identified between conditions in either group.

**TABLE 5:** Results of the LMM for N400 onset latency estimated using fractional area latency.

Fixed effects	Est/beta	SE	*z*	*χ* ^2^	*p*	*η*_*p*_^2^
(Intercept)	208.16	1.48	141.01	—	—	—
Group	6.33	2.95	2.15	4.44	**0.035**	0.07
Masker‐1	−0.65	2.47	−0.26	0.07	0.793	0.00
Masker‐2	0.15	3.35	0.04	0.00	0.965	0.00
Group × Masker‐1	1.79	4.93	0.36	0.13	0.717	0.00
Group × Masker‐2	−6.87	6.69	−1.03	1.04	0.307	0.02

*Note:* The *p*‐values of significant fixed effects are presented in bold. Model structure: lmer(Latency ~1 + Group × Masker‐1 + Group × Masker‐2 + (1 + Masker‐1 + Masker‐2 | Subject)).

**FIGURE 5:**
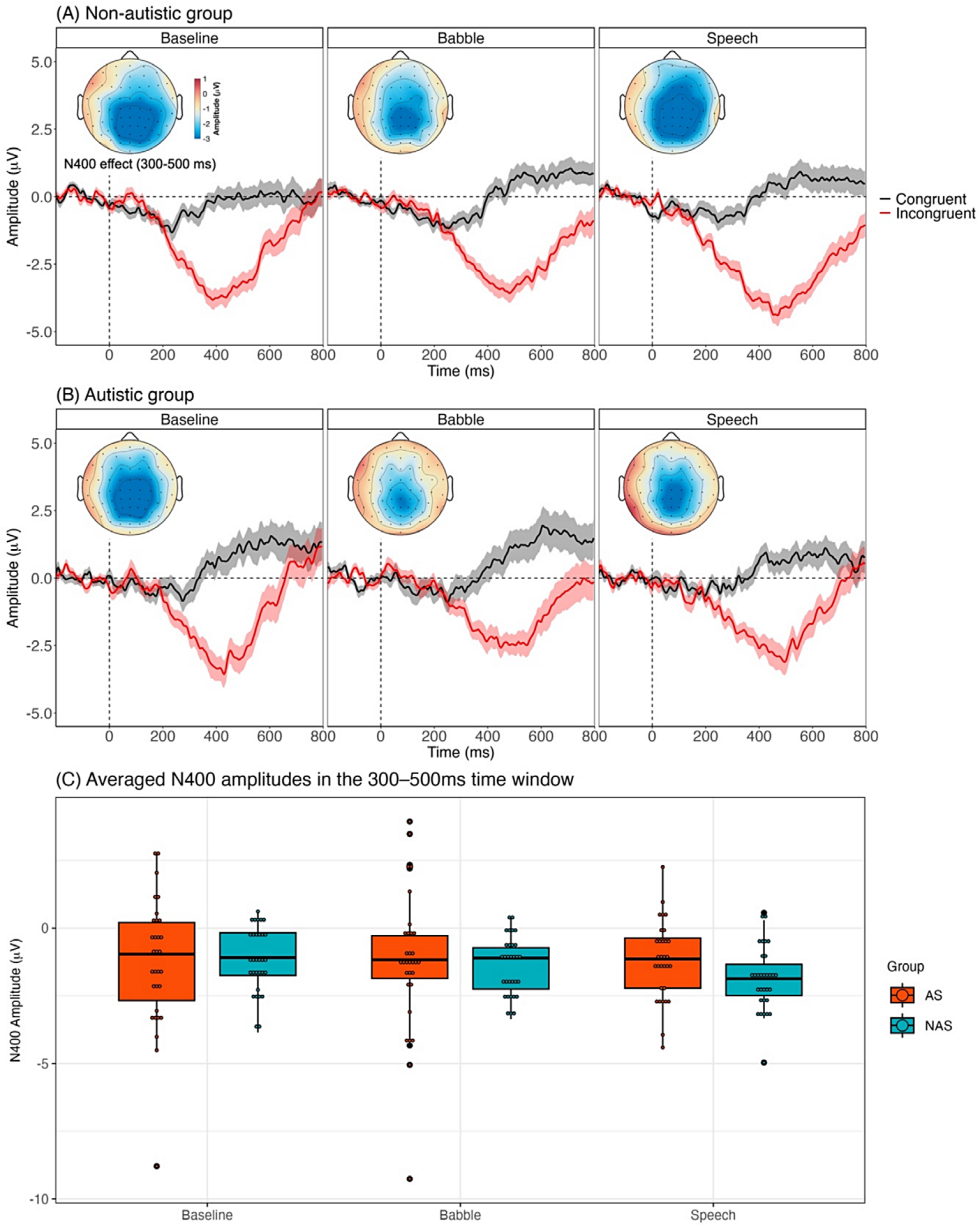
N400 amplitudes across groups and masker conditions. (A, B) ERP waveforms for the non‐autistic (A) and autistic (B) groups, showing mean amplitudes for congruent (black) and incongruent (red) sentences across baseline, babble, and speech masker conditions. Shaded areas represent ±1 SEM. Topographic maps display the spatial distribution of the N400 effect (incongruent minus congruent) averaged across the 300–500 ms time window. (C) Averaged N400 amplitudes (300–500 ms window) for each condition (baseline, babble, and speech) and group (AS, autistic; NAS, non‐autistic).

Then, LMMs were conducted on N400 amplitudes within 300–500 ms to assess between‐group differences across conditions (see Figure 5 for the results). The mean amplitudes from five midline electrodes (Fz, FCz, Cz, CPz, and Pz) were used as the dependent variable (Song et al. 2020). As summarized in Table 6, significant three‐way interactions were found between group, sentence type, and the two masker contrasts.

**TABLE 6:** Results of the LMM for N400 amplitudes.

Fixed effects	Est/beta	SE	*t*	*χ* ^2^	*p*	*η*_*p*_^2^
(Intercept)	−0.56	0.11	−4.91	—	—	
Group	0.53	0.23	2.32	5.14	**0.023**	0.08
Sentence	1.36	0.17	7.92	43.36	**< 0.001**	0.50
Masker‐1	−0.02	0.01	−2.48	6.13	**0.013**	0.00
Masker‐2	0.21	0.01	25.09	628.90	**< 0.001**	0.00
Group × Masker‐1	−0.02	0.01	−1.54	2.36	0.125	0.00
Group × Masker‐2	−0.03	0.02	−1.71	2.91	0.088	0.00
Group × Sentence	−0.25	0.34	−0.74	0.54	0.463	0.01
Masker‐1 × Sentence	0.23	0.01	16.20	206.28	**< 0.001**	0.00
Masker‐2 × Sentence	−0.24	0.02	−14.45	208.77	**< 0.001**	0.00
Group × Masker‐1 × Sentence	−0.56	0.03	−19.57	382.80	**< 0.001**	0.00
Group × Masker‐2 × Sentence	0.54	0.03	16.24	263.71	**< 0.001**	0.00

*Note:* The *p*‐values of significant effects are presented in bold. Model structure: lmer(Amplitude ~1 + Group × Sentence × Masker‐1 + Group × Sentence × Masker‐2 + (1 + Sentence | Subject)).

To better understand the three‐way interactions, we conducted post hoc analyses focusing on two key comparisons: (1) between‐group differences in N400 effect within each masker condition and (2) within‐group N400 effect across different masker conditions. Overall, there were no significant group differences in the N400 effect for any condition, indicating comparable N400 amplitudes between the autistic and non‐autistic groups. However, condition effects were observed only within the non‐autistic group. Specifically, they exhibited a significantly larger N400 response in the masker conditions compared to the baseline condition (*χ* ^2^(1) = 770.25, *p* < 0.001), as well as a significantly larger N400 in the speech condition relative to the babble condition (*χ* ^2^(1) = 582.56, *p* < 0.001). In contrast, no significant condition effects were observed in the autistic group (see Supporting Information for details).

### 3.4 Correlation

To investigate the relationships among cognitive abilities, neural measures of auditory and semantic processing, and task performance, we conducted Pearson correlation analyses for each group and condition. Each analysis included six individual difference measures, including (1) years of professional musical training; (2) Raven's standard score (non‐verbal IQ); (3) ROWPVT percentile (receptive vocabulary); (4) digit span score (working memory); (5) AQ score; and (6) the summed score of auditory attention difficulty and discomfort. Meanwhile, three task‐related measures were also examined, including behavioral accuracy, TRF amplitude, and N400 amplitude. Behavioral performance was indexed by mean accuracy. Semantic processing was quantified using the mean N400 amplitude between 300 and 500 ms, averaged across five midline electrodes (Fz, FCz, Cz, CPz, and Pz). Auditory processing was measured by the difference between the P2 and N1 components of the TRF response across fronto‐central electrodes. This single TRF index was used instead of separate components for two reasons: to reduce the number of variables in the correlation analysis, and because significant condition and group effects were observed within this time window. In total, nine variables were included in the correlation matrix for each group and condition. To control for multiple comparisons, *p*‐values were adjusted using the False Discovery Rate procedure (Benjamini and Hochberg 1995).

**FIGURE 6:**
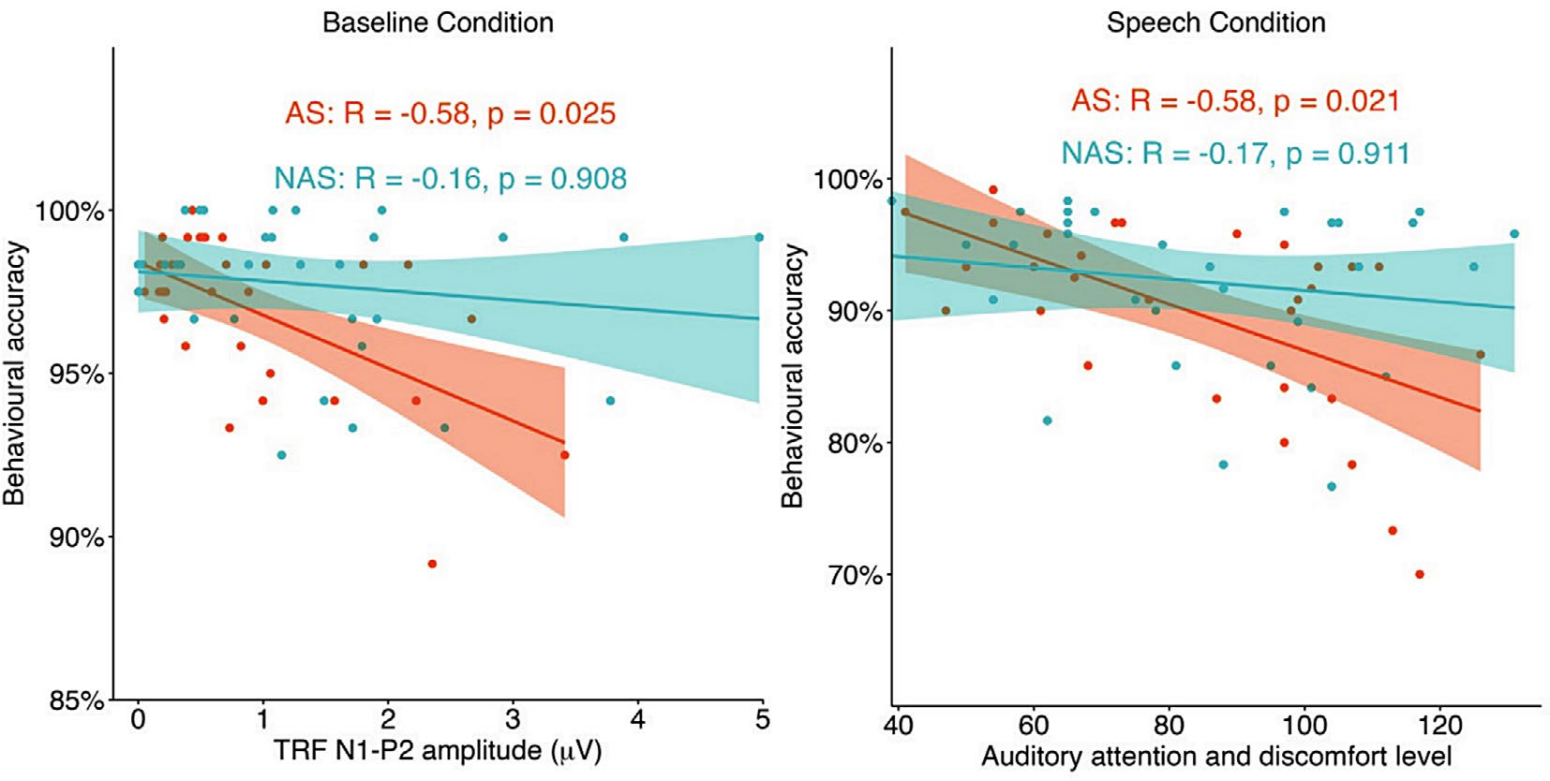
Scatter plots for significant correlations observed in the autistic group (AS) in the baseline and speech condition. The non‐autistic group (NAS) data were plotted for comparison.

Across all conditions and groups, only two significant correlations emerged (see Figure 6). In the baseline condition, a significant negative correlation was found between auditory processing and behavioral performance in the autistic group (*R* = −0.58, *p* = 0.025). Specifically, autistic participants with larger N1–P2 amplitudes tended to show lower behavioral accuracy in response to semantic incongruency. This relationship was not observed in the non‐autistic group. In the speech condition, autistic participants' self‐reported auditory attention difficulty and discomfort score was also negatively correlated with behavioral accuracy (*R* = −0.58, *p* = 0.021), suggesting that those who experience greater auditory challenges in daily life performed more poorly under speech masking. But the relationship was not significant in the non‐autistic group.

## 4 Discussion

This study examined SiN processing in autism by investigating auditory and semantic mechanisms. Although autistic participants showed similar behavioral accuracy and overall N400 amplitudes in response to semantic violations, they exhibited reduced TRF amplitudes, indicating less robust neural encoding of acoustic information, and delayed N400 onset to semantic violations. Moreover, unlike the non‐autistic group, whose neural responses reflected a trade‐off between auditory and semantic processing based on masker type, autistic participants showed no such modulation.

### 4.1 Masker‐Modulated SiN Processing in Non‐Autistic Individuals

To understand how background noise affects auditory processing, we examined TRF components (P1, N1, and P2), which reflect stage‐specific, time‐locked neural responses contributing to speech envelope tracking, along with the *r* value, which quantifies the overall fidelity of neural tracking by measuring how accurately and consistently the brain follows the speech envelope over time.

Compared to the baseline, masker conditions elicited significantly lower TRF *r* values. In our forward modeling, this reflects weaker or less consistent neural tracking of the speech envelope over time, likely due to interference from competing noise. This pattern is consistent with findings from backward modeling studies reporting lower reconstruction accuracy under noisy conditions (Song et al. 2020). Component‐level results offered a more detailed view of processing stages. We found larger P1 amplitudes in masker conditions compared to baseline, consistent with prior studies linking larger P1 responses to degraded speech and louder sounds (Chen et al. 2023; Verschueren et al. 2022). This likely reflects increased demands on early‐stage acoustic encoding due to the presence of interfering sounds. During the N1 time window, masker conditions elicited stronger responses (more negative in amplitude) and longer latencies compared to baseline, reflecting increased attentional engagement. This aligns with evidence showing heightened N1 responses in scenarios requiring greater attention, such as multi‐speaker environments or vocal music processing (Brown and Bidelman 2022; Kong et al. 2014), as well as broader AEP studies linking enhanced N1 amplitude and delayed N1 latency to increased attentional or listening effort (Hillyard et al. 1973). In contrast, P2 amplitudes were reduced in masker conditions relative to baseline. P2 has been widely linked to auditory object formation and speech intelligibility, with larger P2 amplitudes typically associated with better stream segregation and more successful comprehension (Chen et al. 2023; Shinn‐Cunningham et al. 2017). Supporting this, studies in complex auditory scenes have shown that robust P2 responses are linked to successful tracking of the attended speech stream, whereas competing speech often elicits alternative components such as N2 (Fiedler et al. 2019). Thus, the reduction in P2 under noise observed in the current study likely reflects increased difficulty in forming a coherent neural representation of the target speech. These findings align with AEP studies that highlight P2 as a crucial component for forming auditory objects in degraded listening conditions, where larger P2 amplitudes have been associated with more successful segregation of the target speech from background noise (Näätänen and Picton 1987; Strauß et al. 2013).

The comparison between babble and speech conditions revealed no significant differences in *r* values, indicating similar overall neural tracking strength. However, component‐level analyses showed attenuated P1 and N1 amplitudes in the speech masker condition relative to babble, suggesting decreased acoustic encoding and reduced attention orientation for speech maskers. Importantly, this does not necessarily indicate that the babble masker imposes greater auditory demands. As highlighted by Song et al. (2020), babble and speech maskers differ across multiple acoustic and linguistic dimensions, complicating direct comparisons. We therefore follow their approach, viewing these effects as the result of an interplay between semantic and auditory‐level demands in non‐autistic participants.

This interpretation is supported by non‐autistic participants' behavioral and N400 results. Consistent with Song et al. (2020), we found a greater decline in behavioral accuracy in the speech masker condition compared to the babble condition. This was accompanied by larger N400 responses to incongruent words in the speech masker condition, indicating increased semantic processing effort due to greater linguistic interference. Taken together, these findings support the idea of a trade‐off between auditory and semantic processing: when cognitive resources are increasingly allocated to resolving lexical competition under speech masking, fewer may remain available for early auditory encoding. This may account for the reduced early TRF responses (P1/N1) observed in the speech masker condition alongside enhanced semantic engagement (N400) in non‐autistic participants. In conclusion, although different measures of auditory processing were used, our findings in the non‐autistic group closely align with those of Song et al. (2020) and demonstrate the interplay between auditory and semantic processing during SiN listening modulated by masker types.

### 4.2 Atypical Auditory‐Semantic Processing in Autistic Individuals

This is the first study to examine neural tracking of acoustic information in autistic individuals during SiN listening. Our TRF analysis revealed a significant group difference in N1 amplitude, with autistic participants showing reduced responses (i.e., less negative amplitude), as well as a marginally reduced N1–P2 amplitude (i.e., P2 minus N1). These findings are consistent with an AEP study conducted by Teder‐Sälejärvi et al. (2005), who reported flatter N1 spatial gradients in autistic adults during an auditory localization task with competing distractors. These results were interpreted as evidence of a reduced ability to sustain auditory attention in noisy environments. Consistent with this interpretation, previous AEP studies in non‐autistic listeners have shown that attending to speech in noise enhances N1 and P2 amplitudes and shortens their latencies (Billings et al. 2011), whereas reduced motivation and increased listening fatigue are associated with attenuated N1 responses (Moore et al. 2017). Accordingly, the smaller N1 and N1–P2 magnitude we observed in autistic participants may similarly reflect diminished attentional engagement. This neural pattern coincides with behavioral differences: autistic participants reported greater difficulties with auditory attention and sensory sensitivity compared to their non‐autistic peers (see Table 1). Moreover, only in the autistic group did we find a significant relationship between the score of auditory attention difficulty and discomfort (AAD) and behavioral accuracy in the most challenging speech condition. Autistic participants reporting greater everyday auditory challenges (higher AAD scores) performed more poorly when the target speech was presented with competing speech. Together, our findings suggest that background noise may have been more distractive for autistic participants at the acoustic level, making it harder for them to maintain focus and track the target speech stream, especially in more challenging scenarios.

An alternative explanation for group differences in auditory responses comes from a study by Lepistö et al. (2009), which reported that autistic participants showed reduced AEP responses only when processing overlapping auditory streams, but not when the streams were presented separately. This suggests that neural differences may emerge specifically under noisy conditions that place high demands on auditory integration. In our study, cluster‐based permutation tests across the full P1–N1–P2 time window revealed a group difference around the N1 time window in the speech condition. However, because this analysis is exploratory in nature, we followed up with a more targeted statistical approach using LMMs. The LMM analysis revealed a significant main effect of group on TRF N1 amplitude but did not identify any significant group differences within individual conditions. Thus, while the cluster‐based results point to potential group‐specific effects under challenging listening conditions, the statistical analysis does not provide strong evidence that these effects are condition‐specific. This leaves open the question of whether the observed TRF differences are driven by masker complexity or reflect broader group‐level auditory processing differences, even in noise‐free conditions. Further research is needed to clarify how auditory stream integration contributes to SiN difficulties in autism.

Despite significant group differences in TRF amplitudes, *r*‐values did not differ between groups, indicating similar overall encoding accuracy. At first glance, this may seem inconsistent with findings from Jochaut et al. (2015), who reported reduced cortical tracking of the speech envelope in autistic individuals under noise‐free conditions. However, several key methodological differences likely account for this discrepancy. First, the two studies used different approaches to quantify speech tracking. Jochaut et al. linked fMRI responses to the speech envelope to derive spatial tracking indices, which they then cross‐correlated with EEG to assess theta‐band dynamics. In contrast, our study employed forward modeling to estimate TRFs, with *r*‐values reflecting how accurately the speech envelope predicts EEG responses in the time domain. Second, the experimental paradigms differed: Jochaut et al. used naturalistic, paragraph‐length speech in a passive listening task, while our paradigm involved short, semantically manipulated sentences, likely placing lower demands on continuous tracking. Finally, the participant samples varied: the autistic group in Jochaut et al.'s study showed greater variability and generally lower IQ and language abilities, whereas our groups were more closely matched. Overall, our results suggested that while both groups track the speech envelope with comparable precision, they differ in the strength and temporal dynamics of neural encoding, which points to divergent auditory processing mechanisms.

We also offer new insight into SiN processing in autism by examining semantic processing with the N400 component, which has been largely overlooked in previous SiN research. Unexpectedly, unlike most prior studies that found significantly reduced N400 amplitudes and lower behavioral accuracy in noise‐free conditions, our participants showed N400 amplitudes and accuracy comparable to non‐autistic individuals across all the conditions. This is consistent with research demonstrating intact N400 responses to linguistic semantics in autistic adults, despite differences in experimental paradigms (Coderre et al. 2017; O'Rourke and Coderre 2021). One possible explanation for the absence of group difference in N400 amplitudes is the close matching of verbal and cognitive abilities across groups in the current study, which helped control for potential confounding factors that may have influenced results in previous studies (DiStefano et al. 2019; McCleery et al. 2010). Our findings suggest that previously reported N400 differences in autism observed even under less challenging, noise‐free conditions may be largely driven by individual differences in language ability, rather than reflecting a general deficit in semantic processing.

The absence of a group effect may also be attributable to the simplified task design, which reduced semantic demands by manipulating only the final word's congruency in each sentence. The task's predictability may have enabled autistic participants to rely on prior context to anticipate the incongruent word, rather than engaging in deeper semantic processing. This likely contributed to the near‐ceiling behavioral performance observed in both groups, particularly in the baseline condition. As a result, our task might not be sufficiently demanding to detect group differences in semantic processing. Future research could employ more challenging comprehension or decision‐making tasks to better capture variability under noisy listening conditions. We also note that the distribution of N400 amplitudes, particularly in the autistic group (Figure 5), spanned a wider range and included several extreme values, reflecting greater variability across individuals. This variability could have influenced the observed group patterns. However, to reflect the heterogeneity of the autistic population and maintain transparency, we retained all data points, including outliers, in the analysis.

Importantly, although overall N400 amplitudes were comparable between groups, the cluster‐based permutation test revealed a trend toward delayed N400 onset in the autistic group. This delay was accompanied by a more restricted, centrally focused distribution, compared to the broader activation observed in the non‐autistic group during the 200–250 ms time window. Follow‐up analyses of onset latency confirmed a significant group difference, consistent with patterns reported in previous studies under noise‐free conditions (Braeutigam et al. 2008; DiStefano et al. 2019). In the present study, the delayed N400 onset in autistic participants, relative to non‐autistic participants, occurred alongside preserved behavioral accuracy and comparable N400 amplitudes. This suggests that autistic individuals may have required slightly more time or cognitive effort to integrate semantic information to achieve similar outcomes. This interpretation is supported by an eye‐tracking study, which found increased listening effort in autistic children during speech‐in‐noise recognition, despite similar accuracy to non‐autistic peers (Xu et al. 2024).

In summary, we found significantly reduced TRF N1 responses and delayed N400 onset latency across conditions, yet overall similar N400 amplitudes in the autistic group compared with the non‐autistic group. These findings suggest an atypical temporal profile in the auditory‐to‐semantic processing stream in autism. Specifically, the reduced N1 amplitude may reflect diminished attentional engagement or reduced efficiency in encoding acoustic features of speech, while the preserved N400 amplitude indicates that lexical‐semantic integration was ultimately successful. One possibility is that the delayed N400 onset reflects a downstream consequence of atypical early auditory encoding, suggesting that semantic processing was preserved but required more time or effort to compensate for inefficient auditory processing (i.e., reduced TRF N1). Alternatively, as discussed above, the absence of group differences in N400 amplitude may be partly due to the relatively low semantic complexity of the task. From this perspective, the delayed N400 onset may also reflect inefficient semantic processing that was not fully captured by the current paradigm. This interpretation is further supported by the absence of masker‐related modulation effects in the autistic group (see Section 4.3), where N400 amplitudes remained similar across conditions despite varying levels of task difficulty. Future research could further clarify the interaction between auditory and semantic processing in autism by systematically varying both acoustic and semantic demands.

### 4.3 The Absence of Masker‐Modulation in Autistic Individuals

Both groups demonstrated higher accuracy in the babble than in the speech masker condition, indicating behavioral sensitivity to task difficulty. However, only the non‐autistic group exhibited corresponding neural modulation. Specifically, they adjusted their auditory and semantic responses depending on masker type, suggesting a flexible, compensatory strategy that increased semantic processing in response to intelligible background speech. In contrast, the autistic group showed no such modulation at either the auditory or semantic level, suggesting no neural adjustment to listening difficulty. This was evident not only in comparisons across masker types, but also in their N400 responses between baseline and masker conditions. Even in the easier baseline condition—where behavioral performance was near ceiling and significantly better than in masker conditions—the autistic group showed significant N400 amplitudes similar to the masker conditions. This suggests that they engaged similar levels of semantic processing effort regardless of task difficulty. Such a pattern aligns with previous findings of heightened auditory effort under challenging listening demands in autism (Mamashli et al. 2017; Schelinski and Von Kriegstein 2023). One interpretation is that autistic participants may allocate more effort toward processing semantic congruency, potentially at the cost of reduced capacity for top‐down modulation as well as reduced auditory processing. Supporting this, we observed a negative correlation between TRF amplitudes and behavioral accuracy in the baseline condition for the autistic group. Participants with larger auditory responses tended to perform worse behaviorally. Even without background noise, those who showed stronger auditory responses might have fewer cognitive resources available for efficient semantic processing, which resulted in lower behavioral accuracy. However, as we did not observe direct correlations between TRF and N400 amplitudes, the interaction between auditory and semantic processing remains speculative and should be explored further in future studies.

In conclusion, we found that while non‐autistic participants flexibly reallocated cognitive resources between acoustic and semantic processing depending on masker type, no such modulation was observed in the autistic group. These findings suggest that autistic individuals process auditory and semantic information differently in noisy environments, likely due to a combination of differences in sensory encoding and reduced top‐down control. This interpretation is consistent with that of Alcántara et al. (2004), who attributed difficulties in processing speech‐in‐noise with temporal dips to a combination of temporal processing impairments and reduced top‐down modulation. Although no substantial speech recognition difficulties emerged in our controlled task, such atypical processing patterns may limit autistic individuals' ability to adapt in unpredictable or demanding real‐world environments, where effective communication often relies on flexible processing strategies and the integration of bottom‐up and top‐down information (Başkent and Gaudrain 2016; Shinn‐Cunningham and Best 2008).

### 4.4 The Effect of Individual Factors on SiN Processing

Unlike many previous studies on SiN processing in autism, which often involved smaller samples and did not control for between‐group differences in verbal and cognitive abilities (Ruiz Callejo and Boets 2023), our study matched autistic and non‐autistic participants on age, nonverbal IQ, vocabulary, working memory, and musical background. These factors have all been identified in prior research as potential contributors to performance in SiN tasks (Carroll et al. 2016; Gordon‐Salant and Cole 2016; Heinrich and Knight 2016; Rönnberg et al. 2010). Although this group matching approach may limit the generalizability of our findings to the broader autistic population, it allowed us to minimize potential confounds and examine SiN processing within a more defined subgroup. Importantly, the presence of group differences even among autistic individuals with typical verbal and cognitive abilities suggests that their SiN difficulties are not solely due to general language or cognitive abilities but may instead reflect differences in listening strategies or processing patterns under varying conditions.

The only unmatched factor between groups was auditory attention and discomfort (AAD) scores, with the autistic group reporting significantly higher levels of attention difficulties and noise sensitivity. To assess whether this group difference in AAD scores influenced neural or behavioral responses, we conducted complementary (G)LMM analyses for behavioral accuracy, TRF measures, and the N400 component. In these models, the AAD score was included as a covariate, while the fixed and random effects structures remained identical to those used in the main analyses. For both the TRF and N400 models, there were no significant main effects of AAD, and the inclusion of AAD scores did not alter the observed group effects or group‐by‐condition interactions (see Supporting Information for details). This indicates that group‐level differences in AAD scores did not substantially influence neural responses. In contrast, the behavioral accuracy model revealed a significant main effect of AAD: participants with higher AAD scores showed lower accuracy. However, the group effect remained non‐significant, consistent with the original model without the AAD covariate. This suggests that individual differences in auditory attention and discomfort may contribute to variability in behavioral performance, independent of diagnostic group. Additionally, within the autistic group, we found a significant negative correlation between AAD scores and behavioral accuracy in the speech masker condition, indicating that autistic participants with higher AAD scores tended to perform worse in the most challenging listening condition. These findings suggest that while AAD scores do not explain the group‐level neural differences, they may contribute to individual variation in behavioral performance, particularly among autistic individuals in difficult listening conditions.

It should be noted that, although our sample size falls within—or even exceeds—the typical range reported in EEG research (see Clayson et al. 2019 for a discussion), it may still be underpowered to detect subtle group effects given the small effect sizes observed for group‐related differences in both the TRF and N400 data. Future studies with larger and more diverse samples of autistic individuals will be necessary to better characterize the mechanisms underlying speech‐in‐noise processing in autism.

## 5 Conclusion

This is the first EEG study to examine both auditory and semantic level processing of SiN signals in autistic individuals, combining neural tracking measures and N400. The findings highlight distinct auditory and semantic processing between autistic and non‐autistic adults during SiN tasks. Despite similar behavioral accuracy and N400 amplitude, autistic participants showed reduced neural encoding of auditory information, delayed semantic processing, and a lack of modulation by masker type, suggesting differences in processing efficiency and flexibility across multiple levels. These findings contribute to a deeper understanding of SiN processing in autism. Future research could build on these insights to develop strategies that support autistic individuals in noisy social settings, enhancing communication and inclusion.

## Conflicts of Interest

The authors declare no conflicts of interest.

## Supporting information


**Data S1:** Supporting Information.

## Data Availability

The data that support the findings of this study are available from the corresponding author upon reasonable request.

## References

[aur70097-bib-0001] Ahissar, E. , S. Nagarajan , M. Ahissar , A. Protopapas , H. Mahncke , and M. M. Merzenich . 2001. “Speech Comprehension Is Correlated With Temporal Response Patterns Recorded From Auditory Cortex.” Proceedings of the National Academy of Sciences 98, no. 23: 13367–13372. 10.1073/pnas.201400998. PMC60877 11698688

[aur70097-bib-0002] Alcántara, J. I. , E. J. L. Weisblatt , B. C. J. Moore , and P. F. Bolton . 2004. “Speech‐In‐Noise Perception in High‐Functioning Individuals With Autism or Asperger's Syndrome.” Journal of Child Psychology and Psychiatry 45, no. 6: 1107–1114. 10.1111/j.1469-7610.2004.t01-1-00303.x. 15257667

[aur70097-bib-0088] Baron‐Cohen, S. , S. Wheelwright , R. Skinner , J. Martin , and E. Clubley . 2001. “The Autism‐Spectrum Quotient (AQ): Evidence From Asperger Syndrome/High‐Functioning Autism, Males and Females, Scientists and Mathematicians.” Journal of Autism and Developmental Disorders 31, no. 1: 5–17. 10.1023/A:1005653411471. 11439754

[aur70097-bib-0004] Barr, D. J. , R. Levy , C. Scheepers , and H. J. Tily . 2013. “Random Effects Structure for Confirmatory Hypothesis Testing: Keep It Maximal.” Journal of Memory and Language 68, no. 3: 255–278. 10.1016/j.jml.2012.11.001. PMC3881361 24403724

[aur70097-bib-0005] Başkent, D. , and E. Gaudrain . 2016. “Musician Advantage for Speech‐On‐Speech Perception.” Journal of the Acoustical Society of America 139, no. 3: EL51–EL56. 10.1121/1.4942628. 27036287

[aur70097-bib-0092] Bates, D. , M. Maechler , B. Bolker , et al. 2015. “Package ‘lme4’.” Convergence 12, no. 1: 2. http://dk.archive.ubuntu.com/pub/pub/cran/web/packages/lme4/lme4.pdf.

[aur70097-bib-0006] Beauducel, A. , S. Debener , B. Brocke , and J. Kayser . 2000. “On the Reliability of Augmenting/Reducing.” Journal of Psychophysiology 14, no. 4: 226–240. 10.1027//0269-8803.14.4.226.

[aur70097-bib-0093] Benjamini, Y. , and Y. Hochberg . 1995. “Controlling the False Discovery Rate: A Practical and Powerful Approach to Multiple Testing.” Journal of the Royal Statistical Society: Series B (Methodological) 57, no. 1: 289–300. 10.1111/j.2517-6161.1995.tb02031.x.

[aur70097-bib-0007] Bhatara, A. , T. Babikian , E. Laugeson , R. Tachdjian , and Y. S. Sininger . 2013. “Impaired Timing and Frequency Discrimination in High‐Functioning Autism Spectrum Disorders.” Journal of Autism and Developmental Disorders 43, no. 10: 2312–2328. 10.1007/s10803-013-1778-y. 23386117

[aur70097-bib-0084] Bilger, R. C. , J. M. Nuetzel , W. M. Rabinowitz , and C. Rzeczkowski . 1984. “Standardization of a Test of Speech Perception in Noise.” Journal of Speech, Language, and Hearing Research 27, no. 1: 32–48. 10.1044/jshr.2701.32. 6717005

[aur70097-bib-0008] Billings, C. J. , K. O. Bennett , M. R. Molis , and M. R. Leek . 2011. “Cortical Encoding of Signals in Noise: Effects of Stimulus Type and Recording Paradigm.” Ear and Hearing 32, no. 1: 53–60. 10.1097/AUD.0b013e3181ec5c46. 20890206 PMC3010248

[aur70097-bib-0009] Braeutigam, S. , S. J. Swithenby , and A. J. Bailey . 2008. “Contextual Integration the Unusual Way: A Magnetoencephalographic Study of Responses to Semantic Violation in Individuals With Autism Spectrum Disorders.” European Journal of Neuroscience 27, no. 4: 1026–1036. 10.1111/j.1460-9568.2008.06064.x. 18333970

[aur70097-bib-0010] Brodbeck, C. , and J. Z. Simon . 2020. “Continuous Speech Processing.” Current Opinion in Physiology 18: 25–31. 10.1016/j.cophys.2020.07.014. 33225119 PMC7673294

[aur70097-bib-0011] Bronkhorst, A. W. 2000. “The Cocktail Party Phenomenon: A Review of Research on Speech Intelligibility in Multiple‐Talker Conditions.” 86.

[aur70097-bib-0012] Brown, J. A. , and G. M. Bidelman . 2022. “Familiarity of Background Music Modulates the Cortical Tracking of Target Speech at the “Cocktail Party”.” Brain Sciences 12, no. 10: 1320. 10.3390/brainsci12101320. 36291252 PMC9599198

[aur70097-bib-0013] Brungart, D. S. 2001. “Informational and Energetic Masking Effects in the Perception of Two Simultaneous Talkers.” Journal of the Acoustical Society of America 109, no. 3: 1101–1109. 10.1121/1.1345696. 11303924

[aur70097-bib-0095] Carroll, R. , A. Warzybok , B. Kollmeier , and E. Ruigendijk . 2016. “Age‐Related Differences in Lexical Access Relate to Speech Recognition in Noise.” Frontiers in Psychology 7. 10.3389/fpsyg.2016.00990. PMC4930932 27458400

[aur70097-bib-0014] Chen, Y.‐P. , F. Schmidt , A. Keitel , S. Rösch , A. Hauswald , and N. Weisz . 2023. “Speech Intelligibility Changes the Temporal Evolution of Neural Speech Tracking.” NeuroImage 268: 119894. 10.1016/j.neuroimage.2023.119894. 36693596

[aur70097-bib-0015] Clayson, P. E. , K. A. Carbine , S. A. Baldwin , and M. J. Larson . 2019. “Methodological Reporting Behavior, Sample Sizes, and Statistical Power in Studies of Event‐Related Potentials: Barriers to Reproducibility and Replicability.” Psychophysiology 56, no. 11: e13437. 10.1111/psyp.13437. 31322285

[aur70097-bib-0016] Coderre, E. L. , M. Chernenok , B. Gordon , and K. Ledoux . 2017. “Linguistic and Non‐Linguistic Semantic Processing in Individuals With Autism Spectrum Disorders: An ERP Study.” Journal of Autism and Developmental Disorders 47, no. 3: 795–812. 10.1007/s10803-016-2985-0. 28083778

[aur70097-bib-0017] Cohen, J. , P. Cohen , S. G. West , and L. S. Aiken . 2013. Applied Multiple Regression/Correlation Analysis for the Behavioral Sciences. 3rd ed. Routledge. 10.4324/9780203774441.

[aur70097-bib-0018] Crosse, M. J. , G. M. Di Liberto , A. Bednar , and E. C. Lalor . 2016. “The Multivariate Temporal Response Function (mTRF) Toolbox: A MATLAB Toolbox for Relating Neural Signals to Continuous Stimuli.” Frontiers in Human Neuroscience 10: 604. 10.3389/fnhum.2016.00604. 27965557 PMC5127806

[aur70097-bib-0019] Crosse, M. J. , N. J. Zuk , G. M. Di Liberto , A. R. Nidiffer , S. Molholm , and E. C. Lalor . 2021. “Linear Modeling of Neurophysiological Responses to Speech and Other Continuous Stimuli: Methodological Considerations for Applied Research.” Frontiers in Neuroscience 15: 705621. 10.3389/fnins.2021.705621. 34880719 PMC8648261

[aur70097-bib-0020] Delorme, A. , and S. Makeig . 2004. “EEGLAB: An Open Source Toolbox for Analysis of Single‐Trial EEG Dynamics Including Independent Component Analysis.” Journal of Neuroscience Methods 134, no. 1: 9–21. 10.1016/j.jneumeth.2003.10.009. 15102499

[aur70097-bib-0021] DePape, A.‐M. R. , G. B. C. Hall , B. Tillmann , and L. J. Trainor . 2012. “Auditory Processing in High‐Functioning Adolescents With Autism Spectrum Disorder.” PLoS One 7, no. 9: e44084. 10.1371/journal.pone.0044084. 22984462 PMC3440400

[aur70097-bib-0022] Di Liberto, G. M. , J. A. O'Sullivan , and E. C. Lalor . 2015. “Low‐Frequency Cortical Entrainment to Speech Reflects Phoneme‐Level Processing.” Current Biology 25, no. 19: 2457–2465. 10.1016/j.cub.2015.08.030. 26412129

[aur70097-bib-0023] Di Liberto, G. M. , V. Peter , M. Kalashnikova , U. Goswami , D. Burnham , and E. C. Lalor . 2018. “Atypical Cortical Entrainment to Speech in the Right Hemisphere Underpins Phonemic Deficits in Dyslexia.” NeuroImage 175: 70–79. 10.1016/j.neuroimage.2018.03.072. 29609008

[aur70097-bib-0024] Ding, N. , and J. Z. Simon . 2012. “Neural Coding of Continuous Speech in Auditory Cortex During Monaural and Dichotic Listening.” Journal of Neurophysiology 107, no. 1: 78–89. 10.1152/jn.00297.2011. 21975452 PMC3570829

[aur70097-bib-0025] Ding, N. , and J. Z. Simon . 2013. “Adaptive Temporal Encoding Leads to a Background‐Insensitive Cortical Representation of Speech.” Journal of Neuroscience 33, no. 13: 5728–5735. 10.1523/JNEUROSCI.5297-12.2013. 23536086 PMC3643795

[aur70097-bib-0026] DiStefano, C. , D. Senturk , and S. S. Jeste . 2019. “ERP Evidence of Semantic Processing in Children With ASD.” Developmental Cognitive Neuroscience 36: 100640. 10.1016/j.dcn.2019.100640. 30974225 PMC6763343

[aur70097-bib-0027] Dunlop, W. A. , P. G. Enticott , and R. Rajan . 2016. “Speech Discrimination Difficulties in High‐Functioning Autism Spectrum Disorder Are Likely Independent of Auditory Hypersensitivity.” Frontiers in Human Neuroscience 10: 401. 10.3389/fnhum.2016.00401. 27555814 PMC4977299

[aur70097-bib-0028] Emmons, K. A. , A. K. C. Lee , A. Estes , et al. 2022. “Auditory Attention Deployment in Young Adults With Autism Spectrum Disorder.” Journal of Autism and Developmental Disorders 52, no. 4: 1752–1761. 10.1007/s10803-021-05076-8. 34013478 PMC8860962

[aur70097-bib-0029] Fiedler, L. , M. Wöstmann , S. K. Herbst , and J. Obleser . 2019. “Late Cortical Tracking of Ignored Speech Facilitates Neural Selectivity in Acoustically Challenging Conditions.” NeuroImage 186: 33–42. 10.1016/j.neuroimage.2018.10.057. 30367953

[aur70097-bib-0030] Fishman, I. , A. Yam , U. Bellugi , A. Lincoln , and D. Mills . 2011. “Contrasting Patterns of Language‐Associated Brain Activity in Autism and Williams Syndrome.” Social Cognitive and Affective Neuroscience 6, no. 5: 630–638. 10.1093/scan/nsq075. 20802091 PMC3190203

[aur70097-bib-0031] Gillis, M. , L. Decruy , J. Vanthornhout , and T. Francart . 2022. “Hearing Loss Is Associated With Delayed Neural Responses to Continuous Speech.” European Journal of Neuroscience 55, no. 6: 1671–1690. 10.1111/ejn.15644. 35263814

[aur70097-bib-0032] Gordon‐Salant, S. , and S. S. Cole . 2016. “Effects of Age and Working Memory Capacity on Speech Recognition Performance in Noise Among Listeners With Normal Hearing.” Ear and Hearing 37, no. 5: 593–602. 10.1097/AUD.0000000000000316. 27232071

[aur70097-bib-0033] Groen, W. B. , L. Van Orsouw , N. T. Huurne , et al. 2009. “Intact Spectral but Abnormal Temporal Processing of Auditory Stimuli in Autism.” Journal of Autism and Developmental Disorders 39, no. 5: 742–750. 10.1007/s10803-008-0682-3. 19148738

[aur70097-bib-0034] Hagoort, P. 2008. “The Fractionation of Spoken Language Understanding by Measuring Electrical and Magnetic Brain Signals.” Philosophical Transactions of the Royal Society, B: Biological Sciences 363, no. 1493: 1055–1069. 10.1098/rstb.2007.2159. PMC2606796 17890190

[aur70097-bib-0035] Heinrich, A. 2021. “The Role of Cognition for Speech‐In‐Noise Perception: Considering Individual Listening Strategies Related to Aging and Hearing Loss.” International Journal of Behavioral Development 45, no. 5: 382–388. 10.1177/0165025420914984.

[aur70097-bib-0089] Heinrich, A. , and S. Knight . 2016. “The Contribution of Auditory and Cognitive Factors to Intelligibility of Words and Sentences in Noise.” In Physiology, Psychoacoustics and Cognition in Normal and Impaired Hearing, 37–45. Springer International Publishing. https://library.oapen.org/bitstream/handle/20.500.12657/27935/1/1002064.pdf#page=63. 10.1007/978-3-319-25474-6_5 27080644

[aur70097-bib-0036] Henderson, L. M. , H. A. Baseler , P. J. Clarke , S. Watson , and M. J. Snowling . 2011. “The N400 Effect in Children: Relationships With Comprehension, Vocabulary and Decoding.” Brain and Language 117, no. 2: 88–99. 10.1016/j.bandl.2010.12.003. 21272930

[aur70097-bib-0037] Hernandez, L. M. , S. A. Green , K. E. Lawrence , et al. 2020. “Social Attention in Autism: Neural Sensitivity to Speech Over Background Noise Predicts Encoding of Social Information.” Frontiers in Psychiatry 11: 343. 10.3389/fpsyt.2020.00343. 32390890 PMC7194032

[aur70097-bib-0038] Hillyard, S. A. , R. F. Hink , V. L. Schwent , and T. W. Picton . 1973. “Electrical Signs of Selective Attention in the Human Brain.” Science 182, no. 4108: 177–180. 10.1126/science.182.4108.177. 4730062

[aur70097-bib-0039] Holdgraf, C. R. , J. W. Rieger , C. Micheli , S. Martin , R. T. Knight , and F. E. Theunissen . 2017. “Encoding and Decoding Models in Cognitive Electrophysiology.” Frontiers in Systems Neuroscience 11: 61. 10.3389/fnsys.2017.00061. 29018336 PMC5623038

[aur70097-bib-0086] Jochaut, D. , K. Lehongre , A. Saitovitch , et al. 2015. “Atypical Coordination of Cortical Oscillations in Response to Speech in Autism.” Frontiers in Human Neuroscience 9. 10.3389/fnhum.2015.00171. PMC4376066 25870556

[aur70097-bib-0085] Kalikow, D. N. , K. N. Stevens , and L. L. Elliott . 1977. “Development of a Test of Speech Intelligibility in Noise Using Sentence Materials With Controlled Word Predictability.” Journal of the Acoustical Society of America 61, no. 5: 1337–1351. 10.1121/1.381436. 881487

[aur70097-bib-0040] Kong, Y.‐Y. , A. Mullangi , and N. Ding . 2014. “Differential Modulation of Auditory Responses to Attended and Unattended Speech in Different Listening Conditions.” Hearing Research 316: 73–81. 10.1016/j.heares.2014.07.009. 25124153 PMC4194271

[aur70097-bib-0041] Kutas, M. , and S. A. Hillyard . 1980. “Reading Senseless Sentences: Brain Potentials Reflect Semantic Incongruity.” Science 207, no. 4427: 203–205. 10.1126/science.7350657. 7350657

[aur70097-bib-0042] Lau, B. K. , K. A. Emmons , A. K. C. Lee , J. Munson , S. R. Dager , and A. M. Estes . 2023. “The Prevalence and Developmental Course of Auditory Processing Differences in Autistic Children.” Autism Research 16, no. 7: 1413–1424. 10.1002/aur.2961. 37376987 PMC11620991

[aur70097-bib-0043] Lepistö, T. , A. Kuitunen , E. Sussman , et al. 2009. “Auditory Stream Segregation in Children With Asperger Syndrome.” Biological Psychology 82, no. 3: 301–307. 10.1016/j.biopsycho.2009.09.004. 19751798 PMC2771139

[aur70097-bib-0044] Lopez‐Calderon, J. , and S. J. Luck . 2014. “ERPLAB: An Open‐Source Toolbox for the Analysis of Event‐Related Potentials.” Frontiers in Human Neuroscience 8: 213. 10.3389/fnhum.2014.00213. 24782741 PMC3995046

[aur70097-bib-0045] Luck, S. J. 2005. “Ten Simple Rules for Designing and Interpreting ERP Experiments.” In Event‐Related Potentials: A Methods Handbook, 4. MIT Press.

[aur70097-bib-0046] Luo, H. , and D. Poeppel . 2007. “Phase Patterns of Neuronal Responses Reliably Discriminate Speech in Human Auditory Cortex.” Neuron 54, no. 6: 1001–1010. 10.1016/j.neuron.2007.06.004. 17582338 PMC2703451

[aur70097-bib-0047] Mamashli, F. , S. Khan , H. Bharadwaj , et al. 2017. “Auditory Processing in Noise Is Associated With Complex Patterns of Disrupted Functional Connectivity in Autism Spectrum Disorder.” Autism Research 10, no. 4: 631–647. 10.1002/aur.1714. 27910247 PMC5473512

[aur70097-bib-0048] Maris, E. , and R. Oostenveld . 2007. “Nonparametric Statistical Testing of EEG‐ and MEG‐Data.” Journal of Neuroscience Methods 164, no. 1: 177–190. 10.1016/j.jneumeth.2007.03.024. 17517438

[aur70097-bib-0049] Martin, N. A. , and R. Brownell . 2011. Expressive One‐Word Picture Vocabulary Test‐4 (EOWPVT‐4). Academic Therapy Publications.

[aur70097-bib-0050] McClannahan, K. S. , K. C. Backer , and K. L. Tremblay . 2019. “Auditory Evoked Responses in Older Adults With Normal Hearing, Untreated, and Treated Age‐Related Hearing Loss.” Ear and Hearing 40, no. 5: 1106–1116. 10.1097/AUD.0000000000000698. 30762601 PMC6689468

[aur70097-bib-0051] McCleery, J. P. , R. Ceponiene , K. M. Burner , J. Townsend , M. Kinnear , and L. Schreibman . 2010. “Neural Correlates of Verbal and Nonverbal Semantic Integration in Children With Autism Spectrum Disorders.” Journal of Child Psychology and Psychiatry 51, no. 3: 277–286. 10.1111/j.1469-7610.2009.02157.x. 20025622

[aur70097-bib-0052] Moore, T. M. , A. P. Key , A. Thelen , and B. W. Y. Hornsby . 2017. “Neural Mechanisms of Mental Fatigue Elicited by Sustained Auditory Processing.” Neuropsychologia 106: 371–382. 10.1016/j.neuropsychologia.2017.10.025. 29061491 PMC5707129

[aur70097-bib-0053] Muncke, J. , I. Kuruvila , and U. Hoppe . 2022. “Prediction of Speech Intelligibility by Means of EEG Responses to Sentences in Noise.” Frontiers in Neuroscience 16: 876421. 10.3389/fnins.2022.876421. 35720724 PMC9198593

[aur70097-bib-0054] Näätänen, R. , and T. Picton . 1987. “The N1 Wave of the Human Electric and Magnetic Response to Sound: A Review and an Analysis of the Component Structure.” Psychophysiology 24, no. 4: 375–425. 10.1111/j.1469-8986.1987.tb00311.x. 3615753

[aur70097-bib-0055] O'Connor, K. 2012. “Auditory Processing in Autism Spectrum Disorder: A Review.” Neuroscience and Biobehavioral Reviews 36, no. 2: 836–854. 10.1016/j.neubiorev.2011.11.008. 22155284

[aur70097-bib-0056] Oostenveld, R. , P. Fries , E. Maris , and J.‐M. Schoffelen . 2011. “FieldTrip: Open Source Software for Advanced Analysis of MEG, EEG, and Invasive Electrophysiological Data.” Computational Intelligence and Neuroscience 2011, no. 1: 156869. 10.1155/2011/156869. 21253357 PMC3021840

[aur70097-bib-0057] Orf, M. , M. Wöstmann , R. Hannemann , and J. Obleser . 2023. “Target Enhancement but Not Distractor Suppression in Auditory Neural Tracking During Continuous Speech.” iScience 26, no. 6: 106849. 10.1016/j.isci.2023.106849. 37305701 PMC10251127

[aur70097-bib-0058] O'Rourke, E. , and E. L. Coderre . 2021. “Implicit Semantic Processing of Linguistic and Non‐Linguistic Stimuli in Adults With Autism Spectrum Disorder.” Journal of Autism and Developmental Disorders 51, no. 8: 2611–2630. 10.1007/s10803-020-04736-5. 33547603 PMC8254724

[aur70097-bib-0059] Osterhout, L. , and P. J. Holcomb . 1992. “Event‐Related Brain Potentials Elicited by Syntactic Anomaly.” Journal of Memory and Language 31, no. 6: 785–806. 10.1016/0749-596X(92)90039-Z.

[aur70097-bib-0060] Ouimet, T. , N. E. V. Foster , A. Tryfon , and K. L. Hyde . 2012. “Auditory‐Musical Processing in Autism Spectrum Disorders: A Review of Behavioral and Brain Imaging Studies.” Annals of the New York Academy of Sciences 1252, no. 1: 325–331. 10.1111/j.1749-6632.2012.06453.x. 22524375

[aur70097-bib-0061] Pfordresher, P. Q. , and A. R. Halpern . 2013. “Auditory Imagery and the Poor‐Pitch Singer.” Psychonomic Bulletin & Review 20, no. 4: 747–753. 10.3758/s13423-013-0401-8. 23413013

[aur70097-bib-0062] Pijnacker, J. , B. Geurts , M. Van Lambalgen , J. Buitelaar , and P. Hagoort . 2010. “Exceptions and Anomalies: An ERP Study on Context Sensitivity in Autism.” Neuropsychologia 48, no. 10: 2940–2951. 10.1016/j.neuropsychologia.2010.06.003. 20542048

[aur70097-bib-0063] Pion‐Tonachini, L. , K. Kreutz‐Delgado , and S. Makeig . 2019. “ICLabel: An Automated Electroencephalographic Independent Component Classifier, Dataset, and Website.” NeuroImage 198: 181–197. 10.1016/j.neuroimage.2019.05.026. 31103785 PMC6592775

[aur70097-bib-0091] R Core Team . 2022. R: A Language and Environment for Statistical Computing. R Foundation for Statistical Computing. https://www.R‐project.org.

[aur70097-bib-0064] Raven, J. C. , and J. H. Court . 1998. Raven's Progressive Matrices and Vocabulary Scales. Oxford Psychologists Press. http://www.v‐psyche.com/doc/IQ/Raven‐Vocabulary.doc.

[aur70097-bib-0090] Rönnberg, J. , M. Rudner , T. Lunner , and A. Zekveld . 2010. “When Cognition Kicks in: Working Memory and Speech Understanding in Noise.” Noise and Health 12, no. 49: 263. 10.4103/1463-1741.70505. 20871181

[aur70097-bib-0065] Ruiz Callejo, D. , and B. Boets . 2023. “A Systematic Review on Speech‐In‐Noise Perception in Autism.” Neuroscience & Biobehavioral Reviews 154: 105406. 10.1016/j.neubiorev.2023.105406. 37797728

[aur70097-bib-0066] Ruiz Callejo, D. , J. Wouters , and B. Boets . 2023. “Speech‐In‐Noise Perception in Autistic Adolescents With and Without Early Language Delay.” Autism Research 16, no. 9: 1719–1727. 10.1002/aur.2966. 37318057

[aur70097-bib-0067] Russo, N. , S. Zecker , B. Trommer , J. Chen , and N. Kraus . 2009. “Effects of Background Noise on Cortical Encoding of Speech in Autism Spectrum Disorders.” Journal of Autism and Developmental Disorders 39, no. 8: 1185–1196. 10.1007/s10803-009-0737-0. 19353261 PMC2810203

[aur70097-bib-0087] Sassenhagen, J. , and D. Draschkow . 2019. “Cluster‐Based Permutation Tests of MEG/EEG Data Do Not Establish Significance of Effect Latency or Location.” Psychophysiology 56, no. 6: e13335. 10.1111/psyp.13335. 30657176

[aur70097-bib-0068] Schaeffer, J. , M. Abd El‐Raziq , E. Castroviejo , et al. 2023. “Language in Autism: Domains, Profiles and Co‐Occurring Conditions.” Journal of Neural Transmission 130, no. 3: 433–457. 10.1007/s00702-023-02592-y. 36922431 PMC10033486

[aur70097-bib-0069] Schelinski, S. , and K. Von Kriegstein . 2020. “Brief Report: Speech‐In‐Noise Recognition and the Relation to Vocal Pitch Perception in Adults With Autism Spectrum Disorder and Typical Development.” Journal of Autism and Developmental Disorders 50, no. 1: 356–363. 10.1007/s10803-019-04244-1. 31583624

[aur70097-bib-0070] Schelinski, S. , and K. Von Kriegstein . 2023. “Responses in Left Inferior Frontal Gyrus Are Altered for Speech‐In‐Noise Processing, but Not for Clear Speech in Autism.” Brain and Behavior: A Cognitive Neuroscience Perspective 13, no. 2: e2848. 10.1002/brb3.2848. PMC9927852 36575611

[aur70097-bib-0071] Schwartz, S. , L. Wang , S. Uribe , B. G. Shinn‐Cunningham , and H. Tager‐Flusberg . 2023. “Auditory Evoked Potentials in Adolescents With Autism: An Investigation of Brain Development, Intellectual Impairment, and Neural Encoding.” Autism Research 16, no. 10: 1859–1876. 10.1002/aur.3003. 37735966 PMC10676753

[aur70097-bib-0072] Shinn‐Cunningham, B. , V. Best , and A. K. C. Lee . 2017. “Auditory Object Formation and Selection.” In The Auditory System at the Cocktail Party, edited by J. C. Middlebrooks , J. Z. Simon , A. N. Popper , and R. R. Fay , vol. 60, 7–40. Springer International Publishing. 10.1007/978-3-319-51662-2_2.

[aur70097-bib-0073] Shinn‐Cunningham, B. G. , and V. Best . 2008. “Selective Attention in Normal and Impaired Hearing.” Trends in Amplification 12, no. 4: 283–299. 10.1177/1084713808325306. 18974202 PMC2700845

[aur70097-bib-0074] Song, J. , L. Martin , and P. Iverson . 2020. “Auditory Neural Tracking and Lexical Processing of Speech in Noise: Masker Type, Spatial Location, and Language Experience.” Journal of the Acoustical Society of America 148, no. 1: 253–264. 10.1121/10.0001477. 32752786

[aur70097-bib-0075] Strauß, A. , S. A. Kotz , and J. Obleser . 2013. “Narrowed Expectancies Under Degraded Speech: Revisiting the N400.” Journal of Cognitive Neuroscience 25, no. 8: 1383–1395. 10.1162/jocn_a_00389. 23489145

[aur70097-bib-0076] Stringer, L. , and P. Iverson . 2020. “Non‐Native Speech Recognition Sentences: A New Materials Set for Non‐Native Speech Perception Research.” Behavior Research Methods 52, no. 2: 561–571. 10.3758/s13428-019-01251-z. 31012064 PMC7148274

[aur70097-bib-0094] Teder‐Sälejärvi, W. A. , K. L. Pierce , E. Courchesne , and S. A. Hillyard . 2005. “Auditory Spatial Localization and Attention Deficits in Autistic Adults.” Cognitive Brain Research 23, no. 2–3: 221–234. 10.1016/j.cogbrainres.2004.10.021. 15820630

[aur70097-bib-0077] Verschueren, E. , M. Gillis , L. Decruy , J. Vanthornhout , and T. Francart . 2022. “Speech Understanding Oppositely Affects Acoustic and Linguistic Neural Tracking in a Speech Rate Manipulation Paradigm.” Journal of Neuroscience 42, no. 39: 7442–7453. 10.1523/JNEUROSCI.0259-22.2022. 36041851 PMC9525161

[aur70097-bib-0078] Wechsler, H. , T. E. Nelson , J. E. Lee , M. Seibring , C. Lewis , and R. P. Keeling . 2003. “Perception and Reality: A National Evaluation of Social Norms Marketing Interventions to Reduce College Students' Heavy Alcohol Use.” Journal of Studies on Alcohol 64, no. 4: 484–494. 10.15288/jsa.2003.64.484. 12921190

[aur70097-bib-0083] Xu, S. , H. Zhang , J. Fan , et al. 2024. “Auditory Challenges and Listening Effort in School‐Age Children With Autism: Insights From Pupillary Dynamics During Speech‐in‐Noise Perception.” Journal of Speech, Language, and Hearing Research 67, no. 7: 2410–2453. 10.1044/2024_JSLHR-23-00553. 38861391

[aur70097-bib-0080] Yasmin, S. , V. C. Irsik , I. S. Johnsrude , and B. Herrmann . 2023. “The Effects of Speech Masking on Neural Tracking of Acoustic and Semantic Features of Natural Speech.” Neuropsychologia 186: 108584. 10.1016/j.neuropsychologia.2023.108584. 37169066

[aur70097-bib-0081] Zhang, X. , J. Li , Z. Li , et al. 2023. “Leading and Following: Noise Differently Affects Semantic and Acoustic Processing During Naturalistic Speech Comprehension.” NeuroImage 282: 120404. 10.1016/j.neuroimage.2023.120404. 37806465

[aur70097-bib-0082] Zion Golumbic, E. M. , N. Ding , S. Bickel , et al. 2013. “Mechanisms Underlying Selective Neuronal Tracking of Attended Speech at a “Cocktail Party”.” Neuron 77, no. 5: 980–991. 10.1016/j.neuron.2012.12.037. 23473326 PMC3891478

