# Supplementary material for: Auditory and Semantic Processing of Speech‐in‐Noise in Autism: A Behavioral and EEG Study
Source: Autism Res. 2025 Aug 4;18(10):2011–30. doi: 10.1002/aur.70097 (PMC12541682; doi:10.1002/aur.70097)
Supplement: Supplementary file 1 — Data S1: Supporting Information. [file AUR-18-2011-s001.docx]

**Auditory and Semantic Processing of Speech-in-Noise in Autism:**

**A Behavioural and EEG Study**

***Supplementary Material***

# 1. Stimuli

The masker stimuli were adopted from (Song et al., 2020). To prevent participants from relying on acoustic cues to differentiate between competing voices, maskers were created using recordings of the same English stories, read by the same speaker who recorded the target sentences.

For the single-talker speech masker, pauses in the recordings were reduced to less than 25 milliseconds, and low-frequency amplitude modulations (<1 Hz) were attenuated through filtering. In contrast, the babble masker was constructed by removing speaker-specific acoustic features. The stories were segmented into 1.5 to 2.5 second intervals (excluding those with over 15% silence) and spliced together in random order, ensuring the babble remained acoustically uniform and unintelligible.

The signal-to-noise ratio (SNR) level was set to 0 dB based on a pilot study. In this study, six native English speakers participated in this pilot study. Participants were presented with target sentences under four SNR conditions (6 dB, 3 dB, 0 dB, and -3 dB) and were asked to judge sentence acceptability, with accuracy recorded. Supplementary table 1 shows the results of the pilot study. At 6 dB and 3 dB, accuracy rates exceeded 90%. At 0 dB, accuracy dropped to 88.1% in the babble condition and 85.2% in the single-talker condition. At -3 dB, accuracy declined further to 75.6% (babble) and 78.9% (single speaker), with participants reporting significant difficulty hearing the target sentences. Based on these results, 0 dB was selected as the SNR level for the main experiment to prevent ceiling effects while ensuring participants could process linguistic information effectively.

**Supplementary Table 1**. Accuracy rate in the pilot study across conditions and SNR levels.

| Condition | SNR level | Mean (SD) |
| --- | --- | --- |
| Babble | 6 dB | 94.4% (22.9%) |
|  | 3 dB | 92.2% (26.8%) |
|  | 0 dB | 88.1% (32.4%) |
|  | -3 dB | 75.6% (43.1%) |
| Speech | 6 dB | 94.4% (22.9%) |
|  | 3 dB | 90.4% (29.6%) |
|  | 0 dB | 85.2% (35.6%) |
|  | -3 dB | 78.9% (40.1%) |

# 2. Post-hoc analysis for the three-way interaction for the N400 analysis.

To better understand the three-way interaction, we conducted post-hoc analyses focusing on two key comparisons: (1) between-group differences in N400 effect within each masker condition and (2) within-group N400 effect across different masker conditions.

**Group × Sentence Interaction.** We first examined the group-by-sentence interaction in each condition*.* No significant interaction between group and sentence type (α = .025) was observed in the baseline condition (*χ²*(1) = 0.07, *p* = .792), babble condition (χ²(1) = 0.12, *p* = .730) or speech condition (χ²(1) = 4.51, *p* = 0.034). These results indicate that there were no group differences in N400 amplitude in any conditions.

**Masker × Sentence Interaction.** Next, we examined masker-by-sentence interaction in each group. In the non-autistic group, a significant interaction between sentence type and masker contrast 1 (baseline vs. maskers) was observed (*χ²*(1) = 770.25, *p* < .001), while this interaction was marginally significant for autistic participants (*χ²*(1) = 4.84, *p* = .027). Simple effects analyses within the non-autistic group revealed significant differences between baseline and masker conditions for both congruent (*χ²*(1) = 433.15, *p* < .001) and incongruent sentences (*χ²*(1) = 350.58, *p* < .001). Specifically, the congruent condition showed larger amplitudes in the baseline compared to masker conditions, while the incongruent condition showed the opposite, with masker conditions eliciting larger amplitudes than the baseline. This pattern suggests that the presence of maskers amplified the N400 effect in the non-autistic group, a trend not observed in the autistic group. For masker contrast 2 (babble vs. speech), a significant interaction was observed in the non-autistic group (*χ²*(1) = 582.56, p < .001) but not in the autistic group (*χ²*(1) = 1.42, p = .234). Follow-up analyses in the non-autistic group showed no significant difference between the babble and speech conditions for congruent sentences (*χ²*(1) = 5.42, *p =* .020). However, a significant difference was found for incongruent sentences (*χ²*(1) = 861.84, *p* < .001), with the speech condition eliciting larger amplitudes than the babble condition. These findings suggest that while the non-autistic group exhibited a more pronounced N400 effect in the speech condition compared to the babble condition, no similar masker effect was detected in the autistic group.

**3. Complementary (G)LMM Analyses**

Because the two groups differed significantly in their Auditory Attention and Discomfort (AAD) scores—a potential confounding factor influencing both behavioural accuracy and neural responses—we conducted complementary (G)LMM analyses to assess whether this group difference affected our results. Specifically, we re-ran the models for behavioural accuracy, TRF measures (the peak amplitude and latency for TRF components, and model fit r-value), and N400 responses (amplitude and onset latency), this time including AAD score as a covariate. The fixed and random effects structures remained identical to those used in the main analyses. Full results are provided in Supplementary Tables 2–6.

**Supplementary Table 2.** Results of the GLMM for behavioural data including AAD score as a covariate.

| Fixed effects | Est/Beta | SE | z | *χ^2^* | *p* | | OR |
| --- | --- | --- | --- | --- | --- | --- | --- |
| (Intercept) | 4.49 | 0.36 | 12.30 | — | — | | — |
| Group | -0.33 | 0.18 | -1.80 | 3.17 | 0.075 | | 0.72 |
| Masker-1 | -1.13 | 0.12 | -9.34 | 58.85 | **<.001** | | 0.32 |
| Masker-2 | 0.47 | 0.09 | 5.37 | 21.56 | **<.001** | | 1.61 |
| Group × Masker-1 | 0.08 | 0.22 | 0.36 | 0.10 | 0.752 | | 1.07 |
| Group × Masker-2 | 0.12 | 0.16 | 0.76 | 0.61 | 0.434 | | 1.13 |
| AAD score | -0.01 | 0.00 | -2.82 | 7.56 | **0.006** | 0.99 | |
| *Note.* The *p-*values of significant fixed effects are presented in bold. Model structure: glmer(Accuracy ~ 1 + Group × Masker-1 + Group × Masker-2 + (1 + Masker-1 + Masker-2 \| Subject) + (1 \| Trial)). OR: Odds ratios. | | | | | | | |

**Supplementary Table 3**. Results of the LMM for TRF component amplitudes and latency including AAD score as a covariate.

|  | **Fixed effects** | **Est/Beta** | **SE** | **t** | ***χ^2^*** | ***p*** | ***η_p_^2^*** |
| --- | --- | --- | --- | --- | --- | --- | --- |
|  | (Intercept) | 0.66 | 0.15 | 4.27 | — | — | — |
|  | Group | 0.06 | 0.10 | 0.59 | 0.38 | 0.537 | 0.01 |
| P1 | Masker-1 | 0.20 | 0.05 | 3.86 | 13.36 | **<.001** | 0.19 |

| amplitude | Masker-2 | 0.35 | 0.06 | 5.80 | 26.84 | **<.001** | 0.35 |
| --- | --- | --- | --- | --- | --- | --- | --- |
|  | Group × Masker-1 | 0.08 | 0.10 | 0.79 | 0.61 | 0.433 | 0.01 |
|  | Group × Masker-2 | 0.05 | 0.12 | 0.45 | 0.20 | 0.656 | 0.00 |
|  | AAD score | 0.00 | 0.00 | -0.83 | 0.67 | 0.413 | 0.01 |
|  | (Intercept) | 77.66 | 3.60 | 21.597 | — | — | — |
|  | Group | 1.70 | 1.96 | 0.87 | 0.74 | 0.389 | 0.01 |
| P1 | Masker-1 | -1.95 | 1.69 | -1.15 | 1.31 | 0.252 | 0.02 |
| layency | Masker-2 | -2.82 | 1.82 | -1.55 | 2.35 | 0.125 | 0.04 |
|  | Group × Masker-1 | 2.72 | 3.38 | 0.81 | 0.65 | 0.421 | 0.01 |
|  | Group × Masker-2 | -1.73 | 3.64 | -0.48 | 0.23 | 0.635 | 0.00 |
|  | AAD score | -0.04 | 0.04 | -1.01 | 0.95 | 0.331 | 0.02 |
|  | (Intercept) | -0.21 | 0.14 | -1.50 | — | — | — |
|  | Group | 0.16 | 0.08 | 2.13 | 4.26 | **0.039** | 0.07 |
| N1 | Masker-1 | -0.21 | 0.07 | -3.26 | 9.78 | **0.002** | 0.15 |
| amplitude | Masker-2 | -0.22 | 0.05 | -4.13 | 15.05 | **<.001** | 0.22 |
|  | Group × Masker-1 | 0.04 | 0.13 | 0.31 | 0.09 | 0.759 | 0.00 |
|  | Group × Masker-2 | -0.04 | 0.11 | -0.42 | 0.17 | 0.678 | 0.00 |
|  | AAD score | 0.00 | 0.00 | -0.79 | 0.59 | 0.444 | 0.00 |
|  | (Intercept) | 168.78 | 7.98 | 21.15 | — | — | — |
|  | Group | 7.84 | 4.35 | 1.80 | 3.13 | 0.077 | 0.05 |
| N1 | Masker-1 | 19.43 | 2.98 | 6.52 | 32.33 | **<.001** | 0.41 |
| latency | Masker-2 | -1.21 | 3.01 | -0.40 | 0.16 | 0.687 | 0.00 |
|  | Group × Masker-1 | -5.75 | 5.96 | -0.96 | 0.92 | 0.337 | 0.01 |
|  | Group × Masker-2 | -7.34 | 6.01 | -1.22 | 1.47 | 0.225 | 0.02 |
|  | AAD score | 0.02 | 0.09 | 0.25 | 3.16 | 0.075 | 0.00 |
|  | (Intercept) | 0.13 | 0.15 | 0.88 | — | — | — |
|  | Group | -0.08 | 0.10 | -0.76 | 0.57 | 0.451 | 0.01 |
| P2 | Masker-1 | -0.53 | 0.06 | -8.63 | 48.95 | **<.001** | 0.55 |
| amplitude | Masker-2 | -0.11 | 0.05 | -2.18 | 4.59 | **0.032** | 0.07 |
|  | Group × Masker-1 | 0.35 | 0.12 | 2.79 | 7.35 | **0.007** | 0.11 |
|  | Group × Masker-2 | 0.06 | 0.10 | 0.66 | 0.43 | 0.513 | 0.01 |
|  | AAD score | 0.00 | 0.00 | 0.45 | 0.19 | 0.664 | 0.00 |
|  | (Intercept) | 0.53 | 0.22 | 2.46 | — | — | — |
|  | Group | -0.24 | 0.13 | -1.93 | 3.62 | 0.057 | 0.06 |
| N1-P2 | Masker-1 | -0.32 | 0.09 | -3.52 | 11.27 | **<.001** | 0.17 |
| amplitude | Masker-2 | 0.11 | 0.07 | 1.50 | 2.20 | 0.138 | 0.03 |
|  | Group × Masker-1 | 0.31 | 0.18 | 1.67 | 2.73 | 0.098 | 0.04 |
|  | Group × Masker-2 | 0.11 | 0.15 | 0.73 | 0.53 | 0.465 | 0.01 |
|  | AAD score | 0.00 | 0.00 | -0.08 | 0.00 | 0.935 | 0.00 |
| *Note.* The *p*-values of significant effects are presented in bold. The same model was used for all analyses of amplitude and latency: lmer(Amplitude/Latency ~ 1 + Group × Masker-1 + Group × Masker-2 + (1 + Masker-1 + Masker-2 \| Subject)). | | | | | | | |

**Supplementary Table 4**. Results of the LMM for r values of TRF modelling including AAD score as a covariate.

| **Fixed effects** | **Est/Beta** | **SE** | **z** | ***χ^2^*** | ***p*** | ***η_p_^2^*** |
| --- | --- | --- | --- | --- | --- | --- |
| (Intercept) | 0.09 | 0.02 | 3.66 | — | — | — |
| Group | -0.01 | 0.01 | -0.83 | 2.54 | 0.112 | 0.01 |
| Masker-1 | -0.02 | 0.01 | -2.51 | 58.85 | **<.001** | 0.09 |
| Masker-2 | 0.00 | 0.01 | -0.34 | 21.56 | **<.001** | 0.00 |
| Group × Masker-1 | 0.00 | 0.02 | -0.30 | 0.10 | 0.752 | 0.00 |
| Group × Masker-2 | 0.01 | 0.02 | 0.86 | 0.61 | 0.434 | 0.01 |
| AAD score | 0.00 | 0.00 | 0.49 | 0.69 | 0.408 | 0.00 |
| *Note.* The *p-*values of significant fixed effects are presented in bold. Model structure: lmer(r-value ~ 1 + Group × Masker-1 + Group × Masker-2 + (1 + Masker-1 + Masker-2 \| Subject)). | | | | | | |

**Supplementary Table 5.** Results of the LMM for N400 onset latency including AAD score as a covariate.

| **Fixed effects** | **Est/Beta** | **SE** | **z** | ***χ^2^*** | ***p*** | ***η_p_^2^*** |
| --- | --- | --- | --- | --- | --- | --- |
| (Intercept) | 208.20 | 5.31 | 39.23 | — | — | — |
| Group | 6.33 | 2.96 | 2.14 | 4.43 | **.035** | 0.07 |
| Masker-1 | -0.65 | 2.47 | -0.26 | 0.07 | .793 | 0.00 |
| Masker-2 | 0.15 | 3.35 | 0.04 | 0.00 | .965 | 0.00 |
| Group × Masker-1 | 1.79 | 4.93 | 0.36 | 0.13 | .717 | 0.00 |
| Group × Masker-2 | -6.87 | 6.69 | -1.03 | 1.04 | .307 | 0.02 |
| AAD score | 0.00 | 0.06 | -0.01 | 0.00 | .990 | 0.00 |
| *Note.* The *p-*values of significant fixed effects are presented in bold. Model structure: lmer(Latency ~ 1 + Group × Masker-1 + Group × Masker-2 + (1 + Masker-1 + Masker-2 \| Subject)). | | | | | | |

**Supplementary** **Table 6**. Results of the LMM for N400 amplitude including AAD score as a covariate.

| **Fixed effects** | **Est/Beta** | **SE** | **t** | ***χ^2^*** | ***p*** | ***η_p_^2^*** |
| --- | --- | --- | --- | --- | --- | --- |
| (Intercept) | -0.99 | 0.42 | -2.38 | — | — |  |
| Group | 0.54 | 0.23 | 2.38 | 5.42 | **.020** | 0.08 |
| Sentence | 1.36 | 0.17 | 7.92 | 43.36 | **<.001** | 0.50 |
| Masker-1 | -0.02 | 0.01 | -2.48 | 6.13 | **.013** | 0.00 |
| Masker-2 | 0.21 | 0.01 | 25.09 | 628.9 | **<.001** | 0.00 |
| Group × Masker-1 | -0.02 | 0.01 | -1.54 | 2.36 | .125 | 0.00 |
| Group × Masker-2 | -0.03 | 0.02 | -1.71 | 2.91 | .088 | 0.00 |
| Group × Sentence | -0.25 | 0.34 | -0.74 | 0.54 | .463 | 0.01 |
| Masker-1 × Sentence | 0.23 | 0.01 | 16.20 | 206.28 | **<.001** | 0.00 |
| Masker-2 × Sentence | -0.24 | 0.02 | -14.45 | 208.77 | **<.001** | 0.00 |
| Group × Masker-1 × Sentence | -0.56 | 0.03 | -19.57 | 382.8 | **<.001** | 0.00 |
| Group × Masker-2 × Sentence | 0.54 | 0.03 | 16.24 | 263.71 | **<.001** | 0.00 |
| AAD score | 0.01 | 0.00 | 1.08 | 1.14 | 0.285 | 0.02 |
| *Note.* The *p*-values of significant effects are presented in bold. Model structure: lmer(Amplitude ~ 1 + Group × Sentence × Masker-1 + Group × Sentence × Masker-2 + (1 + Sentence \| Subject)). | | | | | | |

**References**

Song, J., Martin, L., & Iverson, P. (2020). Auditory neural tracking and lexical processing of speech in noise: Masker type, spatial location, and language experience. *The Journal of the Acoustical Society of America*, *148*(1), 253–264. https://doi.org/10.1121/10.0001477
